# Efficacy and safety of primaquine and methylene blue for prevention of *Plasmodium falciparum* transmission in Mali: a phase 2, single-blind, randomised controlled trial

**DOI:** 10.1016/S1473-3099(18)30044-6

**Published:** 2018-06

**Authors:** Alassane Dicko, Michelle E Roh, Halimatou Diawara, Almahamoudou Mahamar, Harouna M Soumare, Kjerstin Lanke, John Bradley, Koualy Sanogo, Daouda T Kone, Kalifa Diarra, Sekouba Keita, Djibrilla Issiaka, Sekou F Traore, Charles McCulloch, Will J R Stone, Jimee Hwang, Olaf Müller, Joelle M Brown, Vinay Srinivasan, Chris Drakeley, Roly Gosling, Ingrid Chen, Teun Bousema

**Affiliations:** a Malaria Research and Training Centre, Faculty of Pharmacy, Medicine, and Dentistry, University of Science, Techniques, and Technologies of Bamako, Bamako, Mali; b Global Health Group, Malaria Elimination Initiative, University of California, San Francisco, CA, USA; c Radboud Institute for Health Sciences, Radboud University Medical Center, Nijmegen, Netherlands; d Department of Epidemiology and Biostatistics, University of California, San Francisco, CA, USA; e MRC Tropical Epidemiology Group, London School of Hygiene & Tropical Medicine, London, UK; f Department of Immunology and Infection, London School of Hygiene & Tropical Medicine, London, UK; g President's Malaria Initiative, Malaria Branch, Centers for Disease Control and Prevention, Atlanta, GA, USA; h Institute of Public Health, Ruprecht-Karls-University, Heidelberg, Germany; i Institute for Health Metrics and Evaluation, University of Washington, Seattle, WA, USA

## Abstract

**Background:**

Primaquine and methylene blue are gametocytocidal compounds that could prevent *Plasmodium falciparum* transmission to mosquitoes. We aimed to assess the efficacy and safety of primaquine and methylene blue in preventing human to mosquito transmission of *P falciparum* among glucose-6-phosphate dehydrogenase (G6PD)-normal, gametocytaemic male participants.

**Methods:**

This was a phase 2, single-blind, randomised controlled trial done at the Clinical Research Centre of the Malaria Research and Training Centre (MRTC) of the University of Bamako (Bamako, Mali). We enrolled male participants aged 5–50 years with asymptomatic *P falciparum* malaria. G6PD-normal participants with gametocytes detected by blood smear were randomised 1:1:1:1 in block sizes of eight, using a sealed-envelope design, to receive either sulfadoxine-pyrimethamine and amodiaquine, sulfadoxine-pyrimethamine and amodiaquine plus a single dose of 0·25 mg/kg primaquine, dihydroartemisinin-piperaquine, or dihydroartemisinin-piperaquine plus 15 mg/kg per day methylene blue for 3 days. Laboratory staff, investigators, and insectary technicians were masked to the treatment group and gametocyte density of study participants. The study pharmacist and treating physician were not masked. Participants could request unmasking. The primary efficacy endpoint, analysed in all infected patients with at least one infectivity measure before and after treatment, was median within-person percentage change in mosquito infectivity 2 and 7 days after treatment, assessed by membrane feeding. This study is registered with ClinicalTrials.gov, number NCT02831023.

**Findings:**

Between June 27, 2016, and Nov 1, 2016, 80 participants were enrolled and assigned to the sulfadoxine-pyrimethamine and amodiaquine (n=20), sulfadoxine-pyrimethamine and amodiaquine plus primaquine (n=20), dihydroartemisinin-piperaquine (n=20), or dihydroartemisinin-piperaquine plus methylene blue (n=20) groups. Among participants infectious at baseline (54 [68%] of 80), those in the sulfadoxine-pyrimethamine and amodiaquine plus primaquine group (n=19) had a median 100% (IQR 100 to 100) within-person reduction in mosquito infectivity on day 2, a larger reduction than was noted with sulfadoxine-pyrimethamine and amodiaquine alone (n=12; −10·2%, IQR −143·9 to 56·6; p<0·0001). The dihydroartemisinin-piperaquine plus methylene blue (n=11) group had a median 100% (IQR 100 to 100) within-person reduction in mosquito infectivity on day 2, a larger reduction than was noted with dihydroartemisinin-piperaquine alone (n=12; −6·0%, IQR −126·1 to 86·9; p<0·0001). Haemoglobin changes were similar between gametocytocidal arms and their respective controls. After exclusion of blue urine, adverse events were similar across all groups (59 [74%] of 80 participants had 162 adverse events overall, 145 [90%] of which were mild).

**Interpretation:**

Adding a single dose of 0·25 mg/kg primaquine to sulfadoxine-pyrimethamine and amodiaquine or 3 days of 15 mg/kg per day methylene blue to dihydroartemisinin-piperaquine was highly efficacious for preventing *P falciparum* transmission. Both primaquine and methylene blue were well tolerated.

**Funding:**

Bill & Melinda Gates Foundation, European Research Council.

## Introduction

Since 2000, scale-up of interventions has led to substantial gains in malaria control across sub-Saharan Africa.^1^ Despite progress, new tools will be needed to control and eliminate malaria.^2^

Several malaria control and elimination strategies target the plasmodium parasite in human beings, including case management, seasonal malaria chemo-prevention, and mass drug administration. Drug combinations used in these strategies do not target the gametocyte stage of the parasite, allowing continued human to mosquito transmission despite clearance of the asexual blood stage infection. WHO recommends addition of a single 0·25 mg/kg dose of primaquine (single low-dose primaquine) to standard artemisinin-based combination therapy to reduce onward transmission of *Plasmodium falciparum* in areas of low malaria transmission and emerging antimalarial drug resistance.^3^ However, important questions remain.

**Research in context**

**Evidence before the study**

We searched ClinicalTrials.gov and PubMed between Sept 18, 2015, and Sept 15, 2017, using the search terms “primaquine” OR “methylene blue” AND “malaria” AND “falciparum”, OR “primaquine” OR “methylene blue” AND “transmission”, OR “primaquine” OR “methylene blue” AND “gametocyte”. No language restrictions were used in the search. Although numerous studies, including randomised controlled trials, systematic reviews, and meta-analyses, have reported on the efficacy and safety of single low-dose primaquine as a *Plasmodium falciparum* gametocytocidal drug, only four efficacy studies used membrane feeding assays, the gold standard for infectivity assays, to assess the transmission-blocking effect of primaquine. None of these studies assessed the efficacy of primaquine combined with sulfadoxine-pyrimethamine plus amodiaquine. One study in Colombia assessed the efficacy of adding a single dose of 0·75 mg/kg primaquine to sulfadoxine-pyrimethamine plus amodiaquine on *P falciparum* gametocyte clearance. Using blood smear microscopy, authors found lower gametocytaemia on days 4 and 8, but no clearance of gametocytes by day 8. Since no direct measurements of membrane feeding assays were done, it is unclear if the persisting gametocytes would have been infectious to mosquitoes. Eight published trials of methylene blue were identified. Two studies assessed the effect of methylene blue with either artesunate, amodiaquine, or artesunate-amodiaquine on gametocytes. Groups receiving methylene blue had lower gametocytaemia by at least day 7. Neither of these studies assessed transmission using membrane feeding assays, nor was methylene blue paired with dihydroartemisinin-piperaquine, one of the major drugs used in community drug interventions, known for its long prophylactic period.

**Added value of this study**

To our knowledge, this is the first study to use the membrane feeding assay to directly quantify the effect of primaquine (partnered with sulfadoxine-pyrimethamine plus amodiaquine) and methylene blue (partnered with dihydroartemisinin-piperaquine) on human to mosquito transmission of *P falciparum*. Our findings show that antimalarial combinations of sulfadoxine-pyrimethamine plus amodiaquine with 0·25 mg/kg primaquine and dihydroartemisinin-piperaquine with a 3-day treatment course of 15 mg/kg methylene blue are safe and block human to mosquito transmission by day 2 post-treatment.

**Implications of all the available evidence**

Potent gametocytocidal drugs primaquine and methylene blue are two promising transmission-blocking tools that can be used for falciparum malaria control and elimination. Future studies should focus on assessment of the community-level effect of adding gametocytocidal drugs to first-line treatments and large-scale, drug-based interventions, including seasonal malaria chemoprophylaxis (sulfadoxine-pyrimethamine plus amodiaquine) and mass drug administration (dihydroartemisinin-piperaquine).

First, whether addition of single low-dose primaquine to non-artemisinin-based combination therapy can prevent *P falciparum* transmission is unclear. The antimalarial combination sulfadoxine-pyrimethamine and amodiaquine has strong treatment and chemoprophylactic effects. Sulfadoxine-pyrimethamine and amodiaquine is the standard drug combination for seasonal malaria chemoprevention and has been highly effective in reducing malaria morbidity and mortality in the Sahel and sub-Sahel regions of Africa, where seasonal malaria chemoprevention is widely implemented.^4^ However, the increased gametocyte production associated with sulfadoxine-pyrimethamine and amodiaquine^5^ might influence its ability to affect malaria transmission. Thus, adding an effective gametocytocidal drug could greatly increase the community-level effect of seasonal malaria chemoprevention.

The second question is whether addition of methylene blue (also known as methylthioninium chloride) to artemisinin-based combination therapy reduces post-treatment transmission potential. Methylene blue has highly potent gametocytocidal effects^6^, ^7^ and, if given with artemisinin-based combination therapy, might provide additional protection against the development and spread of artemisinin resistance.^8^ However, no formal infectivity studies have been done using methylene blue. An artemisinin-based combination therapy of particular interest is dihydroartemisinin-piperaquine, an antimalarial regimen commonly used in mass drug administration.^9^, ^10^, ^11^, ^12^ In areas of high sulfa-doxine-pyrimethamine resistance, dihydroartemisinin-piperaquine could be a promising alternative regimen for seasonal malaria chemoprevention. Dihydro-artemisinin-piperaquine is advantageous in mass treatment settings because of the long chemoprophylactic period offered by piperaquine^11^ and activity against immature *P falciparum* gametocytes, resulting in partial reductions in human to mosquito transmission.^13^, ^14^

Since neither sulfadoxine-pyrimethamine and amodiaquine or dihydroartemisinin-piperaquine target the mature stage of *P falciparum* gametocytes (the parasite stage infectious to mosquitoes), the addition of gametocytocidal drugs to these regimens might prevent transmission shortly after treatment and limit the spread of drug-resistant parasites.

The aim of this study was to establish the safety and efficacy of the addition of single low-dose primaquine to sulfadoxine-pyrimethamine and amodiaquine, and the addition of methylene blue to dihydroartemisinin-piperaquine in reducing human to mosquito transmission among glucose 6-phosphate dehydrogenase (G6PD)-normal male participants in Mali.

## Methods

### Study design and participants

This was a phase 2, single-blind, randomised controlled trial done at the Clinical Research Centre of the Malaria Research and Training Centre (MRTC) of the University of Bamako (Bamako, Mali).

Study participants were recruited from the town of Ouélessébougou, Mali, and its surrounding villages. Malaria transmission in this region is hyperendemic and highly seasonal with peaks from July to November. A study team, comprised of clinicians and lab technicians, met with community leaders, village health workers, and heads of households from each village to explain the study and obtained approval to conduct the study. Before participant screening, village health workers used a door-to-door approach to inform households of the date and location where consenting and screening would take place. Participants were enrolled if they were male, aged 5–50 years, G6PD-normal by Carestart G6PD rapid diagnostic test (Access Bio, Somerset, NJ, USA), had a haemoglobin concentration of at least 10 g/dL, had asymptomatic *P falciparum* malaria at enrolment, had at least two *P falciparum* gametocytes per 500 white blood cells on thick film microscopy (corresponding to at least 30 gametocytes per μL, assuming 8000 white blood cells per μL of blood), and had an absence of other non-*P falciparum* species on blood film. Female participants were excluded to mitigate the risk of haemolysis through incorrect classification of G6PD-deficient female heterozygotes. Participants were also excluded if they had a serious or chronic illness (including signs of severe malaria), weighed 80 kg or more, reported antimalarial use within 7 days of screening, or reported allergies to study drugs.

Participants aged at least 18 years provided written informed consent before screening and enrolment. Parental consent was obtained for participants younger than 18 years, and assent was obtained from children aged 12–17 years.

Ethical approval was granted by the Ethics Committee of the Faculty of Medicine, Pharmacy, and Dentistry of the University of Science, Techniques, and Technologies of Bamako (Bamako, Mali), the Committee on Human Research at the University of California San Francisco (UCSF; San Francisco, CA, USA), and the Research Ethics Committee of the London School of Hygiene & Tropical Medicine (London, UK). For the full study protocol see the appendix. The Centers for Disease Control and Prevention investigator's (JH) involvement was approved by the Office of the Associate Director for Science, Center for Global Health (Atlanta, GA, USA) and her participation was deemed not to constitute engagement with human subjects.

### Randomisation and masking

Patients were individually randomised in a 1:1:1:1 ratio, in block sizes of eight, to receive either sulfadoxine-pyrimethamine and amodiaquine, sulfadoxine-pyrimethamine and amodiaquine with single low-dose primaquine, dihydroartemisinin-piperaquine, or dihydroartemisinin-piperaquine with methylene blue. A UCSF investigator (JMB) randomly generated the treatment assignment using Excel (Microsoft Corporation, Redmond, WA, USA), which was linked to a pre-specified, unique participant ID number. The investigator assembled sealed, opaque envelopes with the participant ID number on the outside and treatment assignment contained within. The site pharmacist in Mali (AM) opened the envelopes and provided treatment. The study assignment was masked to investigators and staff involved in assessing all laboratory outcomes. Insectary technicians were masked to the treatment group and gametocyte density of study participants. The study pharmacist and treating physician were not masked. Participants could ask the study physician which treatment they received.

### Procedures

Upon enrolment and collection of day 0 samples, treatment regimens were administered to participants after a fatty snack (biscuits) given to prevent gastrointestinal side-effects and vomiting (associated with methylene blue in previous trials^15^). All study drugs were administered using weight-based dosing, under direct observation of the study pharmacist (AM). Participants in the sulfadoxine-pyrimethamine and amodiaquine group received a single dose of sulfadoxine-pyrimethamine and 3 days of amodiaquine administered by weight to the nearest half tablet (both drugs were provided by Guilin Pharmaceutical, Shanghai, China). Each sulfadoxine-pyrimethamine tablet contained 500 mg sulfadoxine and 25 mg pyrimethamine, and each amodiaquine tablet contained 150 mg. Participants in the sulfadoxine-pyrimethamine plus primaquine group received sulfadoxine-pyrimethamine, as described previously, combined with 0·25 mg/kg single low-dose primaquine (Sanofi Canada, Laval, QC, Canada). Single low-dose primaquine was given as described previously.^16^ Participants in the dihydroartemisinin-piperaquine group received standard doses of dihydroartemisinin-piperaquine (Eurartesim; Sigma-Tau, Pomezia, Italy) according to the manufacturer's instructions. Participants in the dihydroartemisinin-piperaquine plus methylene blue group received dihydroartemisinin-piperaquine, as described previously, combined with 15 mg/kg methylene blue once daily for three consecutive days (45 mg/kg total). Methylene blue was given as minitablets in prepackaged sachets according to the participant's weight group. The methylene blue minitablets were developed at Düsseldorf University (Düsseldorf, Germany), and produced by Pharbil Waltrop (Waltrop, Germany).^7^ For further details on weight-based dosing of drugs see appendix.

Participants were asked to return to the clinic for follow-up visits on days 1, 2, 3, 7, 14, 28, and 42 after enrolment and were encouraged to visit the MRTC clinic any time they were feeling ill. Access to medical care was provided 24 h per day, 7 days a week, and free transport was provided.

Mosquito infectivity was measured on days 0 (pre-treatment), 2, and 7. Venous blood was collected in heparin tubes stored at 37°C and placed in a membrane feeding system within 1 min of collection. For the first three enrolled participants, about 70 *Anopheles gambiae* mosquitoes were fed on participants' blood for 15–20 min with methods previously described.^17^ Because of low infectivity numbers in these individuals, membrane feeding assays were further optimised to include about 90 mosquitoes and maintained throughout the study. Blood-fed mosquitoes were transported to the insectary in Bamako, Mali where they were kept until dissection on day 7 after feeding. Two independent readers assessed the presence of oocysts in 1% mercurochrome. Discordant readings were resolved by a third independent reader. Haemoglobin was measured before treatment (day 0) and at all follow-up visits using HemoCue (AB Leo Diagnostics, Helsingborg, Sweden).

Measurements of gametocyte density were taken before treatment (day 0), and on days 1, 2, 7, 14, 28, and 42 after enrolment. Gametocyte density was measured using blood smear microscopy and by real-time reverse transcription-PCR (qRT-PCR). Blood slides were stained with Giesma and independently read by expert research microscopists over 500 fields for quantification of gametocytes and asexual parasites. For qRT-PCR quantification of female (*Pfs25*) and male (*PfMGET, Pf3D7_1469900*) gametocyte mRNA, 100 μL of blood was collected in EDTA and transferred into 500 μL of RNAprotect Cell Reagent (Qiagen, Hilden, Germany). Samples were stored at MRTC at −80°C until shipment on dry ice at a controlled temperature (−80°C to −70°C). Total nucleic acids were extracted by MagNAPure LC automated extractor (Total Nucleic Acid Isolation Kit-High Performance; Roche Applied Science, Indianapolis, IN, USA). qRT-PCR was done as previously described using sex-specific trendlines of cultured gametocytes.^18^ Samples were declared gametocyte negative if the estimated gametocyte density was less than 0·01 gametocytes per μL (ie, one gametocyte per 100 μL of blood sample).

### Outcomes

The primary outcome was the median within-person percentage change in mosquito infectivity per group, 2 and 7 days after treatment, assessed through membrane feeding and measured by oocyst prevalence in mosquitoes dissected on day 7 after feeding. We originally planned to use the difference in mean values, but amended the analysis plan to use the median value because a few individuals experienced sharp increases in infectivity after enrolment. We defined mosquito infectivity from the participant to the mosquito as the proportion of dissected mosquitos with oocysts, and a participant was defined as infectious (yes or no) if at least one dissected mosquito had at least one oocyst present.

Secondary outcomes included the prevalence, density, circulation time (days), and sex ratios of female and male gametocytes. Estimates of gametocyte sex ratio were restricted to samples with a total gametocyte density greater than 0·2 gametocytes per μL (20 gametocytes per 100 μL of blood sample).^18^ A complete list of secondary outcomes can be found in the protocol (appendix).

Safety outcomes assessed included the number of adverse events and the mean within-person change in haemoglobin concentration per group after treatment, the proportion of participants who had a greater than 25% decrease in haemoglobin, and the proportion of participants with 8 g/dL or less haemoglobin at any point during follow-up. Adverse events were assessed at each follow-up visit and monitored passively by medical staff. All adverse events were graded by severity (eg, mild, grade 1; moderate, grade 2; or severe, grade 3) according to the study's standard operating procedures and based on the Division of Microbiology and Infectious Diseases (DMID) toxicity tables. Adverse events were further categorised based on whether they were considered serious by the treating physician, and categorised by their causal relationship to the study drugs (unrelated or probably not, possibly, probably, or definitely related). Adverse events were considered causal if they were categorised as either possibly, probably, or definitely related to study drug.

### Statistical analysis

Based on preliminary data from a similar infectivity trial,^16^ sample size calculations assumed that the proportion of individuals that would infect at least one mosquito was 0·79, and that on average the proportion of infected mosquitoes was 0·25, resulting in a probability of infection of 0·79 × 0·25=0·198. Using an SD of 0·24 for the change in proportion of infected mosquitoes before and after treatment estimated from a previous trial using single low-dose primaquine,^16^ we did a sample size calculation to detect a 95% reduction in infectivity (from 0·198 to 0·010) with 80% power and a one-tailed significance level of 0·05. This required 20 individuals per group to compare single low-dose primaquine to its comparator group, after allowing for 10% loss to follow-up. No preliminary data were available on the SD of the change in infectivity before and after methylene blue treatment, and calculations assumed a similar SD of 0·24. Our sample size was not defined to compare the transmission-blocking effects between single low-dose primaquine and methylene blue.

For the primary efficacy outcome, we calculated the median within-person percentage change in infectivity and accompanying IQRs for each group. Positive percentages indicated reductions in infectivity. We used a one-sided non-parametric Wilcoxon rank-sum test for pairwise comparisons of the change in infectivity after treatment (control *vs* treatment group). Our analysis of the change in mosquito infectivity included all randomised participants who had an infectivity measurement before treatment and at least one infectivity measurement after treatment. Additionally, we did an analysis of change in infectivity among the subset of participants who infected at least one mosquito after treatment.

We calculated the mean within-person percentage change in haemoglobin for each group at each follow-up visit. Negative values for within-person percentage change in haemoglobin represented decreases in haemoglobin. We used two-sided *t* tests to compare haemoglobin changes between groups. The number of participants who had an adverse event in each group was compared using a two-sided Fisher's exact test. Analyses of adverse events and haemoglobin changes after treatment were restricted to all randomised participants with at least one follow-up visit.

We used qRT-PCR gametocyte densities to calculate the area under the curve (AUC).^19^ We used simple linear regression models for modelling the gametocytocidal effect of primaquine and methylene blue on log_10_ transformed AUC, adjusting for baseline log_10_ gametocyte density. We estimated gametocyte circulation times using a deterministic compartmental model fitted to qRT-PCR gametocyte densities.^20^ The correlation between mosquito infection rates and gametocyte density was assessed using the Spearman correlation coefficient.

All other comparisons between proportions were done using two-sided tests, and we used Fisher's exact test for between-group comparisons and McNemar's test for within-group comparisons. Wilcoxon's signed rank tests were used for within-group comparisons and Wilcoxon rank-sum tests were used for between-group comparisons. All analyses compared treatment with or without gametocytocidal compounds (ie, sulfadoxine-pyrimethamine and amodiaquine plus primaquine *vs* sulfadoxine-pyrimethamine and amodiaquine alone; and dihydroartemisinin-piperaquine plus methylene blue *vs* dihydroartemisinin-piperaquine alone), unless otherwise stated. Statistical analyses were done using Stata version 14.0 and SAS version 9.4.

The trial was overseen by an independent data safety monitoring committee, monitored by an external clinical trials monitoring group (Agence Africaine de Recherche en Santé Humaine; Dakar, Senegal), and registered with ClinicalTrials.gov (number NCT02831023).

### Role of the funding source

The funders of the study had no role in study design, data collection, data analysis, data interpretation, or writing of the report. The corresponding author had full access to all the data in the study and had final responsibility for the decision to submit the publication.

## Results

**Figure 1:**
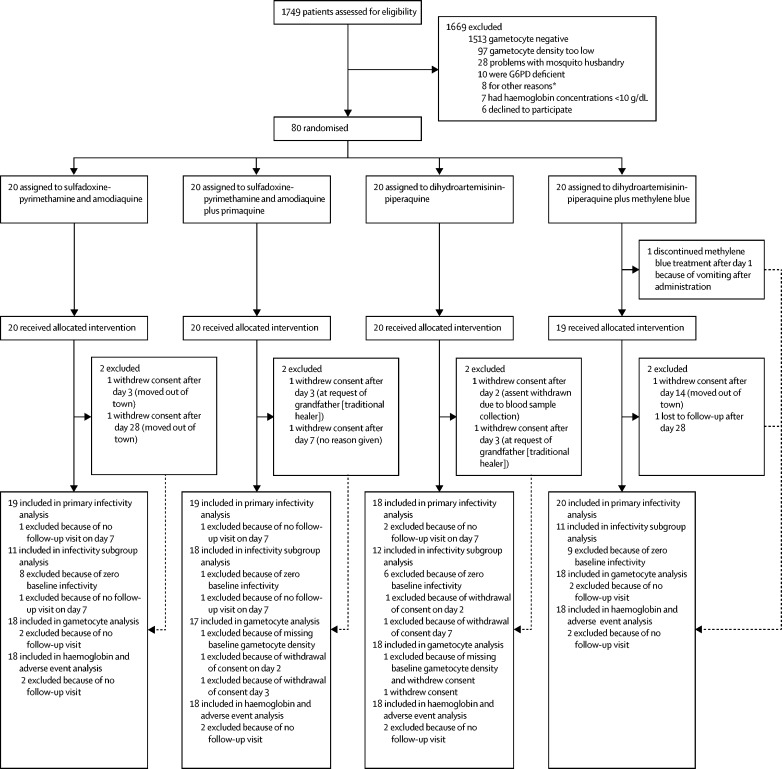
Trial profile *Other reasons included pre-existing chronic disease condition (n=3), recent use of antimalarial drugs (n=1), and no blood smear results (smear deteriorated or lost; n=4).

Between June 27, 2016, and Nov 1, 2016, 1749 male participants were screened for eligibility. 80 participants were enrolled and randomly assigned to receive either sulfadoxine-pyrimethamine and amodiaquine plus primaquine (n=20), dihydroartemisinin-piperaquine plus methylene blue (n=20), and their comparator groups, sulfadoxine-pyrimethamine and amodiaquine alone (n=20), and dihydroartemisinin-piperaquine alone (n=20; figure 1). 72 (90%) of 80 participants completed follow-up to day 42. Baseline characteristics were similar across groups (table 1).

**Table 1:** Baseline characteristics

	**Overall (n=80)**	**Sulfadoxine-pyrimethamine and amodiaquine (n=20)**	**Sulfadoxine-pyrimethamine and amodiaquine plus primaquine (n=20)**	**Dihydroartemisinin-piperaquine (n=20)**	**Dihydroartemisinin-piperaquine plus methylene blue (n=20)**
Age (years)	11 (8·0–14·5)[5–48]	12 (9·0–14·5)[5–48]	10 (7·5–13·0)[5–17]	12·5 (7·5–16·0)[5–35]	10 (8·0–15·0)[5–48]
Weight (kg)	27·5 (22·7–39·7) [14·6–78·5]	28·8 (23·8–39·1) [15·3–78·5]	26·0 (19·7–37·4) [15·9–66·2]	31·1 (21·9–40·5) [16·9–77·2]	26·6 (23·3–40·7)[14·6–69·0]
Primaquine dose (mg/kg per day)	..	..	0·25 (0·24–0·25) [0·23–0·25]	..	..
Methylene blue dose (mg/kg per day)*	..	..	..	..	15·0 (13·9–15·7)[11·6–16·9]
Haemoglobin (g/dL)	12·0 (11·3–12·9) [10·0–16·7]	12·3 (11·5–13·4) [10·0–16·7]	11·7 (10·9–12·3) [10·2–13·6]	12·2 (11·3–13·0) [10·0–14·4]	12·2 (12·2–11·5)[10·0–14·4]
Gametocyte density by microscopy (per μL)	48 (32–80)	48 (32–72)	72 (32–96)	48 (16–64)	48 (32–56)
Asexual parasite prevalence by microscopy	45 (56%)	12 (60%)	12 (60%)	10 (50%)	11 (55%)
Asexual parasite density by microscopy (per μL)	293 (133–1040)	533 (200–1320)	187 (53–400)	493 (213–2720)	400 (133–2427)

Data are median (IQR) or n (%) unless otherwise indicated. Ranges are given in square brackets.

* Given over 3 days.

**Figure 2:**
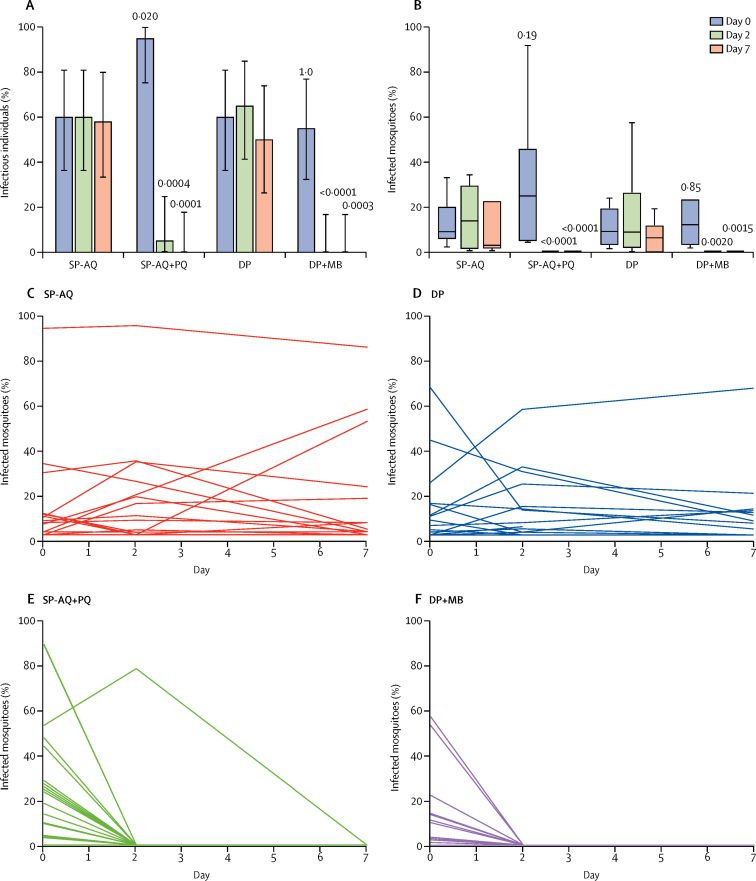
Mosquito infectivity outcomes by treatment group and visit (A) Proportion of infectious individuals. Bars indicate 95% CIs. (B) Proportion of infected mosquitoes among those infectious at baseline. The line indicates the median, the box indicates the 25th–75th quartiles, and the whiskers indicate the highest and lowest values. p values represent testing for between-group differences. Within-person change in mosquito infectivity in the (C) SP-AQ, (D) DP, (E) SP-AQ + PQ, and (F) DP + MB treatment groups. Each line represents the change in the proportion of mosquitoes that were infected from membrane feeding assays done on each individual on days 0, 2, and 7. The proportion of infected mosquitoes was defined by the number of mosquitoes with oocysts present divided by the total number of mosquitoes that survived up to the day of dissection. SP-AQ=sulfadoxine-pyrimethamine and amodiaquine. PQ=primaquine. DP=dihydroartemisinin-piperaquine. MB=methylene blue.

80 membrane feeding assays were done before treatment, 80 on day 2 after treatment, and 76 on day 7 after treatment. 20 599 mosquitoes were used, 18 026 (88%) of which survived to the day of dissection. Before treatment, 54 (68%) of 80 individuals infected at least one mosquito (appendix; figure 2), and 908 (15%) of 6185 mosquitoes became infected (appendix). After treatment, only one (5%) participant in the sulfadoxine-pyrime-thamine and amodiaquine plus primaquine group was infectious at day 2 compared with 12 (60%) patients in the sulfadoxine-pyrimethamine and amodiaquine group, and no patients were infectious at day 7 compared with 11 (58%) of 19 patients in the sulfadoxine-pyrimethamine and amodiaquine group (appendix; figure 2). After treatment, no participants in the dihydroartemisinin-piperaquine plus methylene blue group were infectious on day 2—compared with 13 (65%) patients in the dihydroartemisinin-piperaquine group—or on day 7—compared with nine (50%) of 18 patients in the dihydroartemisinin-piperaquine group (appendix; figure 2). Addition of primaquine or methylene blue was associated with substantial reductions in mosquito infectivity at day 2 and day 7 of treatment compared with control regimens, in both uninfected and infected before treatment populations (table 2; figure 2).

**Table 2:** Median within-person percentage change in mosquito infectivity at day 2 and day 7 after treatment

	**Reduction at day 2**				**Reduction at day 7**			
	n	Median (IQR)	p value*	p value†	n	Median (IQR)	p value*	p value†
**Population assessed for infectivity (n=80)**								
Sulfadoxine-pyrimethamine and amodiaquine	20 (25%)	0% (−19·5 to 4·9)	0·39	ref	19 (24%)	0% (0 to 77·2)	0·17	ref
Sulfadoxine-pyrimethamine and amodiaquine plus primaquine	20 (25%)	100% (100 to 100)	<0·0001	<0·0001	19 (25%)	100% (100 to 100)	<0·0001	<0·0001
Dihydroartemisinin-piperaquine	20 (25%)	0% (−64·9 to 25·4)	0·50	ref	18 (23%)	0% (0 to 92·0)	0·19	ref
Dihydroartemisinin-piperaquine plus methylene blue	20 (25%)	100% (0 to 100)	0·0005	0·0022	20 (25%)	100% (0 to 100)	0·0005	0·033
**Participants infectious before treatment (n=54)**								
Sulfadoxine-pyrimethamine and amodiaquine	12 (22%)	−10·2% (−143·9 to 56·6)	0·39	ref	11 (20%)	42·9% (−1·4 to 95·7)	0·17	ref
Sulfadoxine-pyrimethamine and amodiaquine plus primaquine	19 (35%)	100% (100 to 100)	<0·0001	<0·0001	18 (33%)	100% (100 to 100)	<0·0001	<0·0001
Dihydroartemisinin-piperaquine	12 (22%)	−6·0% (−126·1 to 86·9)	0·50	ref	12	82·9% (−62·1 to 100)	0·19	ref
Dihydroartemisinin-piperaquine plus methylene blue	11 (20%)	100% (100 to 100)	0·0005	<0·0001	11 (20%)	100% (100 to 100)	0·0005	0·0007

* Non-parametric Wilcoxon signed rank test used to assess within-group differences.

† Non-parametric Wilcoxon rank-sum test used to assess between-group differences.

**Figure 3:**
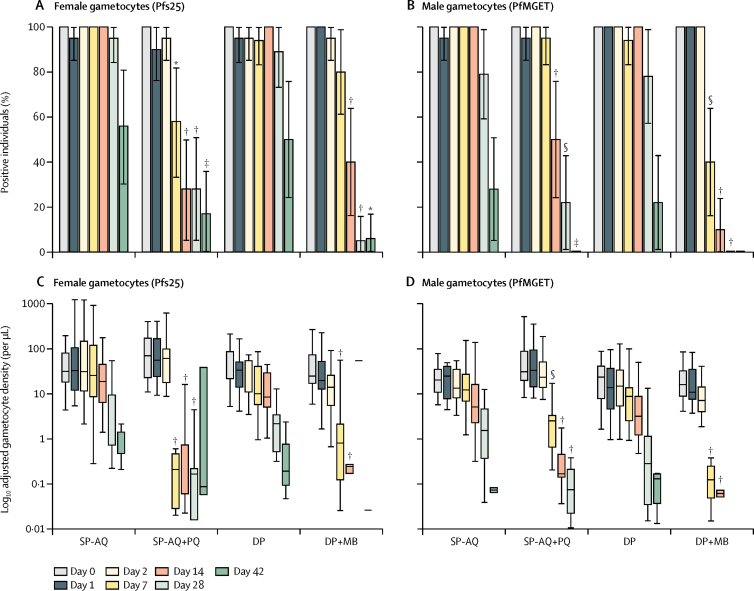
Gametocyte prevalence and density Gametocyte prevalence (A, B) and log_10_ adjusted female (Pfs25) and male (PfMGET) gametocyte densities per μL (C, D) by group and visit. In C and D the line indicates the median, the box indicates the 25th–75th quartiles, and the whiskers indicate the highest and lowest values. *p≤0·01. †p≤0·0001. ‡p≤0·05. §p≤0·001. See appendix for exact p values. SP-AQ=sulfadoxine-pyrimethamine and amodiaquine. PQ=primaquine. DP=dihydroartemisinin-piperaquine. MB=methylene blue.

Before treatment, all study participants with blood samples available for molecular analysis (n=78) had detectable concentrations of both female and male gametocytes. Gametocyte sex ratios were female biased at enrolment, with no differences in gametocyte prevalence or density between study arms (appendix). The prevalence and density of both female and male gametocytes declined during follow-up in all treatment groups (figure 3). This decline was gradual in the sulfadoxine-pyrimethamine and amodiaquine group, whereas estimates were generally lower in the sulfadoxine-pyrimethamine and amodiaquine plus primaquine group from day 7 onwards (figure 3; appendix). Similarly, the prevalence and density of both female and male gametocytes declined more rapidly after dihydroartemisinin-piperaquine plus methylene blue treatment than with dihydroartemisinin-piperaquine alone (figure 3; appendix). The AUC of both female and male gametocyte density over time was lower in both groups that received gametocytocidal drugs than in the groups that did not (appendix).

The mean circulation time of female gametocytes was longer than that of male gametocytes for all treatment arms (appendix). The sulfadoxine-pyrimethamine and amodiaquine plus primaquine treatment group had shorter circulation times for both female and male gametocytes than did the sulfadoxine-pyrimethamine and amodiaquine alone treatment group, as did the dihydroartemisinin-piperaquine plus methylene blue treatment group compared with the dihydroartemisinin-piperaquine treatment group (appendix). Male gametocyte circulation time was decreased in the dihydroartemisinin-piperaquine plus methylene blue group compared with the group receiving sulfadoxine-pyrimethamine and amodiaquine plus primaquine. Gametocyte sex ratios generally became more female biased towards the end of follow-up (appendix). Groups treated with primaquine or methylene blue showed distinct patterns of gametocyte clearance in the first 2 weeks after initiation of treatment. Sex ratio was male biased on day 7 after sulfadoxine-pyrimethamine and amodiaquine plus primaquine treatment (mean female to male gametocyte ratio 0·02) and equal numbers of male and female gametocytes were detected on day 14 (mean ratio 1·0; appendix). By contrast, the very short circulation time of male gametocytes after dihydroartemisinin-piperaquine plus methylene blue treatment resulted in a strongly female-biased sex ratio in this treatment group on day 7 (mean ratio 17·2) and day 14 (mean ratio 7·4; appendix).

Correlations were found between qRT-PCR gametocyte density and mosquito infectivity rates (appendix). Fairly strong correlations were observed in both female and male markers before treatment (appendix). By day 2, correlations remained strong in groups receiving non-gametocytocidal compounds, but had decreased in groups receiving primaquine or methylene blue (appendix).

Absolute and within-person percentage change in mean haemoglobin concentration did not differ between the sulfadoxine-pyrimethamine and amodiaquine plus primaquine and sulfadoxine-pyrimethamine and amodiaquine alone groups or the dihydroartemisinin-piperaquine plus methylene blue and dihydro-artemisinin-piperaquine alone groups at any point during follow-up (appendix). One participant, a 9-year-old boy assigned to the sulfadoxine-pyrimethamine and amodiaquine group, had a more than 25% drop in haemoglobin (from 10 g/dL at baseline to 6 g/dL on day 28). This participant had a positive blood smear on day 26 after treatment with PCR-confirmed recrudescent clinical malaria and was treated with artemether-lumefantrine. The participant's haemoglobin returned to 10·7 g/dL on day 42. No other patients had a haemoglobin lower than 8 g/dL at any timepoint.

Overall, 59 (74%) of 80 participants reported a total of 162 adverse events during follow-up, with 19 of these events being bluish colouration of urine (table 3). The number of participants who had an adverse event differed between the dihydroartemisinin-piperaquine alone and dihydroartemisinin-piperaquine plus methylene blue groups (p=0·031), although this effect was mainly due to frequent reports of blue urine among participants in the methylene blue group. As blue urine is not known to adversely affect health, a separate subanalysis excluding blue urine was done. After exclusion of blue urine, the number of participants that had an adverse event was similar across the four groups. Of the 162 adverse events that occurred throughout the study period, 145 (90%) were mild (grade 1) and 16 (10%) were moderate (grade 2) events. One (1%) severe (grade 3) headache was reported on day 7 in a participant in the sulfadoxine-pyrimethamine and amodiaquine group, although this event was considered unlikely to be caused by study drug. Of the 80 adverse events that were considered related to study drug, 78 (98%) were mild and two (3%) were of moderate severity (headache and loss of appetite). Of the 78 mild adverse events, 19 (24%) were blue urine. An 11-year-old participant in the dihydroartemisinin-piperaquine plus methylene blue arm halted methylene blue treatment after day 1 because of vomiting after drug administration. No other adverse events resulted in stopping of the trial.

**Table 3:** Adverse events

		**Sulfadoxine-pyrimethamine and amodiaquine**	**Sulfadoxine-pyrimethamine and amodiaquine plus primaquine**	**Dihydrosrtemisinin-piperaquine**	**Dihydroartemisinin-piperaquine plus methylene blue**
Participants with an adverse event		17 (85%)	13 (65%)	11 (55%)	18 (90%)
Number of adverse events		51 [20]	31 [15]	30 [16]	50 [29]
Abdominal pain					
	Mild	8 [6]	6 [3]	5 [3]	3 [3]
Bacterial infection					
	Mild	1	0	0	0
	Moderate	0	0	0	1
Belching					
	Mild	1 [1]	0	0	0
Bluish colouration of urine					
	Mild	0	0	0	19 [19]
Bradycardia					
	Mild	0	0	0	1 [1]
Cold					
	Mild	1	0	0	0
Conjunctivitis					
	Mild	0	1	1	0
Cough					
	Mild	0	2	2	2
Diarrhoea					
	Mild	0	0	3 [3]	1
Discomfort					
	Mild	1	1	0	0
	Moderate	1	0	0	0
Dizziness					
	Mild	2	4 [3]	0	1
Dysuria					
	Mild	0	0	1	0
Ear infection					
	Mild	1	0	0	0
Ear pain					
	Moderate	0	0	0	1
Eye pain					
	Mild	0	0	1	0
Fatigue					
	Mild	2	0	0	0
	Moderate	1	0	0	0
Fever					
	Mild	0	0	0	1
	Moderate	1	0	0	0
Headache					
	Mild	6	6 [4]	7 [4]	6 [1]
	Moderate	3 [1]	1	1	2
	Severe	1	0	0	0
Influenza					
	Mild	0	0	1	0
Insomnia					
	Mild	0	1	0	0
Loss of appetite					
	Mild	2 [1]	1	1 [1]	1
	Moderate	1 [1]	0	0	0
Malaria					
	Mild	1	0	0	0
	Moderate	1	0	0	0
Nasal obstruction					
	Mild	0	0	0	1
Nausea					
	Mild	4 [3]	3 [3]	1 [1]	4 [3]
Pain from insect bite					
	Mild	0	0	1	0
Rhinobronchitis					
	Mild	1	0	0	0
Runny nose					
	Mild	1	2 [1]	1	1
Itch					
	Mild	2 [2]	0	0	1
Itchy eyes					
	Mild	0	0	0	1
Shivers					
	Mild	0	1	0	0
Skin rash					
	Mild	1 [1]	0	0	1
Swelling of right hand					
	Moderate	1	0	0	0
Vomiting					
	Mild	6 [4]	1 [1]	4 [4]	2 [2]
	Moderate	0	1	0	0

Numbers of drug-related adverse events are given in square brackets. Drug-related adverse events were defined as possibly, probably, or definitely related to study treatment.

## Discussion

This study shows the pronounced transmission-blocking effects of addition of gametocytocidal drugs to standard antimalarial treatments. Addition of single low-dose primaquine to sulfadoxine-pyrimethamine and amodiaquine or methylene blue to dihydroartemisinin-piperaquine resulted in almost complete blockage of human to mosquito transmission by day 2, and a larger reduction in transmission than that offered by sulfadoxine-pyrimethamine and amodiaquine or dihydroartemisinin-piperaquine alone. No differences in adverse events (excluding blue urine) or haemolysis were observed between groups, suggesting primaquine and methylene blue are safe in this population.

We examined gametocyte dynamics and infectivity to mosquitoes following treatment with sulfadoxine-pyrimethamine and amodiaquine plus primaquine or dihydroartemisinin-piperaquine plus methylene blue, two drug regimens that have important roles in malaria control and elimination efforts. The importance of our direct assessments of infectivity is illustrated by the observation that the transmission-blocking effect of gametocytocidal drugs precedes gametocyte clearance.^21^ Additionally, our findings support observations that infectious gametocytes persist after administration of sulfadoxine-pyrimethamine and amodiaquine^5^ or dihydroartemisinin-piperaquine.^16^, ^22^ Therefore, early identification and treatment of *P falciparum* infection to prevent formation of mature gametocytes is crucial for malaria control.

To our knowledge, this study is the first to formally show the potent transmission-blocking effect of methylene blue in *P falciparum* gametocyte carriers using mosquito infectivity assays, confirming the potency of methylene blue as suggested by previous in-vitro^23^, ^24^ and gametocyte clearance^6^, ^7^ studies. Our study suggests that methylene blue is safe and has a strong gametocytocidal effect when partnered with dihydroartemisinin-piperaquine. However, vomiting has been associated with methylene blue,^15^ and development of blue urine could affect compliance. Future studies are needed to determine the lowest efficacious dose of methylene blue and formulate a more simplified treatment schedule that allows its use as a single-dose gametocytocide, similar to primaquine.^16^ However, a 3-day dose of methylene blue might be more advantageous in the context of artemisinin-based combination therapy resistance because of the broad activity of methylene blue against non-gametocytes.^8^

When combined with sulfadoxine-pyrimethamine and amodiaquine,^5^ single low-dose primaquine had a pronounced effect on *P falciparum* gametocyte carriage and infectivity. Sulfadoxine-pyrimethamine and amodiaquine alone did not have an effect on reducing human to mosquito transmission in the week following treatment. Most individuals treated with sulfadoxine-pyrimethamine and amodiaquine carried gametocytes for at least 1 month following treatment with no reduction in mosquito infection rates achieved by day 7 after treatment. Sulfadoxine-pyrimethamine and amodiaquine is recommended by WHO for use in seasonal malaria chemoprevention. Although the primary objective of seasonal malaria chemoprevention is to reduce malaria morbidity and mortality and not to directly reduce transmission, adding single low-dose primaquine to seasonal malaria chemoprevention might confer indirect community benefits for malaria transmission by reducing post-treatment transmission potential. Transmission reduction by seasonal malaria chemoprevention could be further improved by expanding the age range to include adolescents because gametocyte carriage is high in this population^25^, ^26^ as observed by the age group enrolled in our study.

Countries moving towards malaria elimination could consider adding a transmission-blocking drug to first-line treatments or community-wide mass drug administration to rapidly reduce population-level malaria transmission. Modelling studies suggest that, given adequate coverage and multiple rounds of mass drug administration, addition of gametocytocidal drugs (eg, primaquine and methylene blue) to antimalarial combinations with a long prophylactic period (eg, dihydroartemisinin-piperaquine) might result in larger reductions in transmission than with dihydroartemisinin-piperaquine treatment alone.^27^, ^28^ Formal trials are needed to confirm the best-use scenario for these drug-based transmission-blocking tools, including the target transmission intensity and appropriate integration into other malaria interventions.

Our study revealed that gametocytocidal drugs might have different effects on male and female gametocytes. The strongly reduced circulation time of male gametocytes following methylene blue treatment supports hypotheses generated from in-vitro assessments that methylene blue disproportionally affects male gametocytes.^24^ Although, primaquine results in a rapid reduction in both male and female gametocytes, its early transmission-blocking effect might reflect an early sterilising effect^18^, ^21^ rather than a more rapid clearance of male gametocytes.^18^

Our study has several limitations. First, the study population analysed was not as per intention to treat, as one patient was lost to follow-up and seven patients withdrew. This factor was unlikely to cause attribution bias since the reasons given for withdrawals were unrelated to study drug. Second, our study endpoint was the proportion of oocyst-positive mosquitoes, based on the assumption that even low oocyst densities are likely to render mosquitoes infective.^18^ However, not all mosquitoes with oocysts might have gone on to produce infective sporozoites or could have done so at a reduced rate. Third, the study sample was limited to G6PD-normal male participants in Mali with high *P falciparum* gametocyte densities. Therefore, our findings might not represent the effect of single low-dose primaquine and methylene blue in other malaria-infected populations, particularly those with lower gametocyte densities who have lower pre-treatment and post-treatment transmission potential and G6PD-deficient populations. Although studies in Burkina Faso showed that methylene blue is unlikely to be responsible for clinically relevant haemolysis in individuals with or without G6PD-deficiency,^29^ additional studies are needed on the safety of methylene blue among G6PD-deficient individuals. For single low-dose primaquine, doses from 0·25 mg/kg^30^ to 0·40 mg/kg have been shown to be safe among G6PD-deficient individuals aged 5–50 years.^31^

Adding a single dose of 0·25 mg/kg primaquine to sulfadoxine-pyrimethamine and amodiaquine or 3 days of 15 mg/kg per day methylene blue to dihydroartemisinin-piperaquine were safe and highly efficacious methods for preventing human to host *P falciparum* transmission in G6PD-normal male participants. Further work is needed to understand what effect these findings could have on malaria control and elimination strategies, using mathematical models and community trials to assess costs, risks, and benefits of adding single low-dose primaquine or methylene blue to existing regimens.

For **Division of Microbiology and Infectious Diseases toxicity tables** see https://www.niaid.nih.gov/research/dmid-safetyreporting-pharmacovigilance
