# Supplementary material for: Efficacy and safety of primaquine and methylene blue for prevention of Plasmodium falciparum transmission in Mali: a phase 2, single-blind, randomised controlled trial
Source: Lancet Infect Dis. 2018 Jun;18(6):627–39. doi: 10.1016/S1473-3099(18)30044-6 (PMC5968371; doi:10.1016/S1473-3099(18)30044-6)
Supplement: Supplementary appendix [file mmc1.pdf]

THE LANCET Infectious Diseases

Supplementary webappendix

This webappendix formed part of the original submission and has been peer reviewed. We post it as supplied by the authors.

Supplement to: Dicko A, Roh M E, Diawara H, et al. Efficacy and safety of primaquine and methylene blue for prevention of *Plasmodium falciparum* transmission in Mali: a phase 2, single-blind, randomised controlled trial. *Lancet Infect Dis* 2018; published online Feb 5. http://dx.doi.org/10.1016/S1473-3099(18)30044-6.

SUPPLEMENTARY TABLES

Supplementary Table 1. Weight-based dosing of treatments by day*.

Sulphadoxine-pyrimethamine

Weight (kg)	Number of Fansidar® tablets containing 500mg sulphadoxine and 25 mg pyrimethamine		
	Day 1	Day 2	Day 3
11-20	1	1	1
21-30	1·5	1·5	1·5
31-45	2	2	2
>45	3	3	3

Amodiaquine

Weight (kg)	Number of 150mg tablets		
	Day 1	Day 2	Day 3
15-18	1·5	1	1
19-24	1·5	1·5	1·5
25-35	2·5	2·5	2
36-50	3	3	3
>50	4	4	3

Dihydroartemisinin-piperaquine

Weight (kg)	Dihydroartemisinin (mg)	Piperaquine (mg)	Number of tablets per dose
5 to <7	10	80	½ x 160mg / 20mg tablet
7 to <13	20	160	1 x 160mg / 20mg tablet
13 to <24	40	320	1 x 320mg / 40mg tablet
24 to <36	80	640	2 x 320mg / 40mg tablet
36 to <75	120	960	3 x 320mg / 40mg tablet
75 to 80	160	1,280	4 x 320mg / 40mg tablet

Methylene blue

Weight (kg)	Targeted daily dose (mg) given 1x/day for 3 days
6·0–8·9	100
9·0–12.9	150
13·0–16·9	200
17·0-19·9	250
20·0 – 23·0	300
23.·1 – 29·0	400
29·1 – 36·0	500
36·1 – 43·0	600
43·1 – 50·0	700
50·1 – 80·0	800

* Primaquine was dosed using the following formula: Vol (cc) = 0·25 mg/kg x Weight (kg).

Supplementary Table 2. Results from membrane feeding assay.

	Treatment group	Infectious individuals % (n/N*)	p-value[1]	p-value[2]	Infected mosquitoes % (n/N**)	p-value[3]	p-value[4]	Number of oocysts per mosquito*** Median (range)	p-value[5]	p-value[6]
Day 0	SP-AQ	60 (12/20)	Ref	Ref	11·2 (170/1517)	Ref	Ref	0·03 (0, 0.94)	Ref	Ref
	SP-AQ +PQ	95 (19/20)	Ref	0·020	27·1 (420/1551)	Ref	<0·0001	0·22 (0, 91)	Ref	0·0053
	DP	60 (12/20)	Ref	Ref	10·0 (159/1603)	Ref	Ref	0·02 (0, 0·68)	Ref	Ref
	DP +MB	55 (11/20)	Ref	1·00	10·5 (159/1514)	Ref	0·60	0·02 (0, 0·59)	Ref	0·84
Day 2	SP-AQ	60 (12/20)	1·00	Ref	13·7 (214/1566)	0·043	Ref	0·02 (0, 0·96)	0.74	Ref
	SP-AQ +PQ	5 (1/20)	<0·0001	0·0004	4·3 (65/1512)	<0·0001	<0·0001	0 (0, 0·80)	0·0001	0·0006
	DP	65 (13/20)	0·66	Ref	9·3 (142/1522)	0·59	Ref	0·03 (0, 0·57)	0·87	Ref
	DP +MB	0 (0/20)	0·0001	<0·0001	0 (0/1534)	<0·0001	<0·0001	0 (0, 0)	0·0002	0·0001
Day 7	SP-AQ	58 (11/19)	1·00	Ref	12·2 (168/1370)	0·39	Ref	0·01 (0, 0·86)	0·91	Ref
	SP-AQ +PQ	0 (0/19)	<0·0001	0·0001	0 (0/1510)	<0·0001	<0·0001	0 (0, 0)	0·0001	0·0001
	DP	50 (9/18)	0·38	Ref	8·6 (115/1340)	0·23	Ref	0·01 (0, 0·67)	0·69	Ref
	DP +MB	0 (0/20)	0·001	0·0003	0 (0/1487)	<0·0001	<0·0001	0 (0, 0)	0·0002	0·0004

DP = dihydroartemisinin-piperaquine, MB = methylene blue, PQ = primaquine, SP-AQ = sulphadoxine-pyrimethamine plus amodiaquine
* Proportion of individuals who infected at least 1 mosquito.
** Proportion of infected mosquitoes. N includes mosquitoes from non-infectious individuals.
*** Calculated by taking the total number of oocysts divided by total number of surviving mosquitoes for each individual.
[1] p-value obtained from McNemar's Test to test difference in the proportion of infectious individuals from baseline.
[2] Fisher's exact test to assess whether there is a difference in the proportion of infectious individuals between treatment groups.
[3] Fisher's exact test to assess whether there is a difference in the proportion of infected mosquitoes between visits.
[4] Fisher's exact test to assess whether there is a difference in the proportion of infected mosquitoes between treatment groups.
[5] Non-parametric, Wilcoxon signed rank test to assess within-group differences.
[6] Non-parametric Wilcoxon rank-sum test to assess between-group differences.

Supplementary Table 3. Female and male (Pfs25 and PfMGET) gametocyte density*, prevalence, and sex ratio** by treatment group and visit. Change in area under the curve and gametocyte circulation times assessed by treatment group.

Visit	Gametocyte marker		SP-AQ	SP-AQ +PQ	p-value***	DP	DP +MB	p-value***
Day 0	Pfs25 qRT-PCR	Prevalence % (n/N)	100 (20/20)	100 (19/19)	1·00	100 (19/19)	100 (20/20)	1·00
	Pfs25 qRT-PCR	Density (per mL); median (IQR)	31731 (18240, 8620)	70307 (33961, 172584)	0·13	47753 (21777, 86696)	24932 (17106, 78440)	0·45
	PfMGET qRT-PCR	Prevalence % (n/N)	100 (20/20)	100 (19/19)	1·00	100 (19/19)	100 (20/20)	1·00
	PfMGET qRT-PCR	Density (per mL); median (IQR)	20742 (10851, 35216)	30974 (19907, 88920)	0·14	23550 (7852, 41400)	15996 (8865, 32582)	0·74
	Pfs25:PfMGET ratio	Median (IQR)	2·3 (0·7, 3·7)	1·3 (0·8, 3·6)	0·75	2·2 (1·2, 3·9)	2·1 (1·3, 3·0)	0·61
Day 1	Pfs25 qRT-PCR	Prevalence % (n/N)	95 (19/20)	90 (18/20)	1·00	95 (18/19)	100 (20/20)	0·49
	Pfs25 qRT-PCR	Density (per mL); median (IQR)	32885 (12022, 105925)	56143 (24266, 167494)	0·19	33784 (14093, 50933)	19756 (12907, 54147)	0·86
	PfMGET qRT-PCR	Prevalence % (n/N)	95 (19/20)	95 (19/20)	1·00	100 (19/19)	100 (20/20)	1·00
	PfMGET qRT-PCR	Density (per mL); median (IQR)	24831 (7762, 40832)	33420 (14355, 94406)	0·11	13709 (4592, 36813)	10936 (7568, 35318)	0·88
	Pfs25:PfMGET ratio	Median (IQR)	2·2 (0·8, 3·0)	1·5 (0·9, 3·1)	0·93	1·9 (1·1, 4·3)	2·0 (1·1, 2·8)	0·84
Day 2	Pfs25 qRT-PCR	Prevalence % (n/N)	100 (19/19)	95 (19/20)	1·00	95 (19/20)	95 (19/20)	1·00
	Pfs25 qRT-PCR	Density (per mL); median (IQR)	31189 (11561, 147571)	61235 (17865, 99083)	0·90	18535 (11041, 54576)	13996 (5521, 25882)	0·18
	PfMGET qRT-PCR	Prevalence % (n/N)	100 (19/19)	100 (20/20)	1·00	100 (20/20)	100 (20/20)	1·00
	PfMGET qRT-PCR	Density (per mL); median (IQR)	13366 (8147, 35156)	23421 (13921, 52110)	0·23	14878 (5377, 33777)	7124 (4000, 14263)	0·14
	Pfs25:PfMGET ratio	Median (IQR)	1·8 (1·0, 3·5)	1·6 (1·1, 2·7)	0·65	1·9 (0·8, 3·3)	1·7 (1·0, 2·5)	0·74
Day 7	Pfs25 qRT-PCR	Prevalence % (n/N)	100 (19/19)	58 (11/19)	0·0030	94 (17/18)	80 (16/20)	0·34
	Pfs25 qRT-PCR	Density (per mL); median (IQR)	25586 (8630, 119399)	209 (28, 463)	0·0001	10023 (5794, 40738)	812 (123, 2177)	0·0001
	PfMGET qRT-PCR	Prevalence % (n/N)	100 (19/19)	95 (18/19)	1·00	94 (17/18)	40 (8/20)	0·0010
	PfMGET qRT-PCR	Density (per mL); median (IQR)	12190 (6871, 27290)	2506 (649, 3281)	0·0062	8730 (2523, 13646)	124 (48, 268)	0·0014
	Pfs25:PfMGET ratio	Median (IQR)	2·4 (1·4, 3·8)	0·02 (0·008, 0·1)	<0·0001	2·0 (1·1, 3·6)	17·2 (4·0, 34·8)	0·0070
Day 14	Pfs25 qRT-PCR	Prevalence % (n/N)	100 (19/19)	28 (5/18)	<0·0001	100 (18/18)	40 (8/20)	<0·0001
	Pfs25 qRT-PCR	Density (per mL); median (IQR)	18706 (6471, 45082)	86 (60, 729)	0·014	8539 (5070, 29242)	242 (171, 271)	0·0001
	PfMGET qRT-PCR	Prevalence % (n/N)	100 (19/19)	50 (9/18)	<0·0001	100 (18/18)	10 (2/20)	<0·0001
	PfMGET qRT-PCR	Density (per mL); median (IQR)	5105 (2275, 16181)	165 (140, 447)	0·0001	3168 (1216, 8750)	50, 71****	0·023
	Pfs25:PfMGET ratio	Median (IQR)	2·2 (1·5, 4·1)	1·0 (0·2, 5·5)	0·29	3·1 (1·6, 5·3)	7·4 (4·0, 10·9)	0·21

Visit	Gametocyte marker		SP-AQ	SP-AQ +PQ	p-value***	DP	DP +MB	p-value***
Day 28	Pfs25 qRT-PCR	Prevalence % (n/N)	95 (18/19)	28 (5/18)	<0·0001	89 (16/18)	5 (1/19)	<0·0001
	Pfs25 qRT-PCR	Density (per mL); median (IQR)	2817 (724, 9397)	165 (16, 217)	0·011	2168 (518, 3434)	54702****	0·10
	PfMGET qRT-PCR	Prevalence % (n/N)	79 (15/19)	22 (4/18)	0·0010	78 (14/18)	0 (0/19)	<0·0001
	PfMGET qRT-PCR	Density (per mL); median (IQR)	1528 (366, 4581)	80 (29, 248)	0·021	279 (34, 1127)	0	NA
	Pfs25:PfMGET ratio	Median (IQR)	3·6 (2·4, 6·9)	8·2 (4·6, 11·8)	0·34	7·7 (3·7, 9·2)	NA	NA
Day 42	Pfs25 qRT-PCR	Prevalence % (n/N)	56 (10/18)	17 (3/18)	0·035	50 (9/18)	6 (1/18)	0·0070
	Pfs25 qRT-PCR	Density (per mL); median (IQR)	747 (472, 1413)	57, 86, 38726	0·61	191 (93, 759)	26****	0·12
	PfMGET qRT-PCR	Prevalence % (n/N)	28 (5/18)	0 (0/18)	0·045	22 (4/18)	0 (0/18)	0·10
	PfMGET qRT-PCR	Density (per mL); median (IQR)	72 (63, 81)	0	NA	131 (58, 164)	0	NA
	Pfs25:PfMGET ratio	Median (IQR)	22·4 (10·8, 26·9)	NA	NA	17·4 (7·8, 40·2)	NA	NA
Entire time of follow-up	AUC Pfs25 density/µL versus time	Median (IQR)	9·4 (3·8, 44·2)	4·1 (2·1, 7·9)	0·052	6·2 (2·6, 12·2)	1·6 (0·9, 5·6)	0·013
	Change in AUC of Pfs25	β (95% CI)**	Ref	-0·64 (-0·98, -0·29)	0·0010	Ref	-0·42 (-0·58, -0·27)	<0·0001
	AUC PfMGET density/µL versus time	Median (IQR)	3·3 (2·3, 8·2)	2·4 (1·5, 6·4)	0·40	2·9 (1·1, 6·1)	0·8 (0·5, 2·2)	0·0094
	Change in AUC of PfMGET	β (95% CI)**	Ref	-0·33 (-0·51, -0·15)	0·0010	Ref	-0·38 (-0·61, -0·17)	0·0010

AUC = area under the curve, IQR = interquartile range, CI = confidence interval, DP = dihydroartemisinin-piperaquine, MB = methylene blue, PQ = primaquine, qRT-PCR = quantitative reverse-transcriptase polymerase chain reaction, Ref = reference, SP-AQ = sulphadoxine-pyrimethamine plus amodiaquine

* Densities reported only among gametocyte positive individuals. Thresholds for gametocyte positivity was >0.01 gametocytes/µL.

** Ratios reported only among those who had a total gametocyte density >0·02 gametocytes/mL (i.e. 20 gametocytes in the 100µL blood sample)

*** p-value represents between-group differences. Differences in gametocyte prevalence was tested using the Fisher's exact test. The Wilcoxon rank-sum test was used to test differences in gametocyte density and sex ratios.

**** When ≤3 samples were gametocyte positive, individual gametocyte densities are reported.

Supplementary Table 4. Female and male gametocyte circulation times per treatment arm

Treatment	Female gametocytes (Pfs25)			Male gametocytes (PfMGET)		
	Circulation time in days (95% CI)	Difference in days (95% CI)	p-value*	Circulation time in days (95% CI)	Difference in days (95% CI)	p-value*
SP-AQ	8·6 (7·3-9·8)	Ref		7·3 (6·5-8·2)	Ref	
SP-AQ +PQ	4·4 (3·4-5·5)	4·2 (2·7-5·7)	0·0051	3·2 (2·8-3·5)	4·1 (3·2-5·0)	<0·0001
DP	8·8 (7·7-10·0)	Ref		6·9 (6·2-7·6)	Ref	
DP +MB	3·7 (2·9-4·5)	5·1 (3·8-6·4)	<0·0001	1·7 (1·4-2·0)	5·2 (4·5-5·9)	<0·0001

CI = confidence interval, DP = dihydroartemisinin-piperaquine, MB = methylene blue, PQ = primaquine, Ref = reference, SP-AQ = sulphadoxine-pyrimethamine plus amodiaquine

* p-value represents two sample t-test testing difference in mean circulation time between groups.

Supplementary Table 5. Correlations between mosquito infectivity and log10 adjusted gametocyte density for both female (Pfs25) and male (PfMGET) markers.

		Day 0			Day 2			Day 7		
		n	*r* (95% CI)	p-value*	n	*r* (95% CI)	p-value*	n	*r* (95% CI)	p-value*
Pfs25	Overall	78	0·49 (0·30, 0·64)	<0·0001	76	0·35 (0·13, 0·53)	0·0023	63	0·64 (0·46, 0·77)	<0·0001
	SP-AQ and DP	39	0·46 (0·17, 0·68)	0·0030	38	0·51 (0·23, 0·72)	0·0011	36	0·60 (0·34, 0·78)	0·0001
	SP-AQ +PQ	19	0·41 (-0·05, 0·73)	0·078	19	0·34 (-0·14, 0·85)	0·15	11	NA**	NA
	DP +MB	20	0·50 (0·07, 0·77)	0·025	19	NA**	NA	16	NA**	NA
		Day 0			Day 2			Day 7		
		n	*r* (95% CI)	p-value*	n	*r* (95% CI)	p-value	n	*r* (95% CI)	p-value*
PfMGET	Overall	78	0·58 (0·41, 0·71)	<0·0001	79	0·4 (0·20, 0·57)	0·0020	62	0·62 (0·44, 0·76)	<0·0001
	SP-AQ and DP	39	0·54 (0·27, 0·73)	0·0004	39	0·63 (0·37, 0·79)	<0·0001	36	0·74 (0·54, 0·86)	0·0001
	SP-AQ +PQ	19	0·57 (0·15, 0·81)	0·011	20	0·34 (-0·19, 0·68)	0·14	18	NA**	NA
	DP +MB	20	0·54 (0·13, 0·79)	0·014	8	NA**	NA	20	NA**	NA

CI = confidence interval, DP = dihydroartemisinin-piperaquine, MB = methylene blue, NA = not applicable, PQ = primaquine, *r* = Spearman Correlation coefficient, SP-AQ = sulphadoxine-pyrimethamine plus amodiaquine
* p-value test whether Spearman correlation coefficient is equal to zero.
** No correlations were computed for the DP group on days 2 and 7 or for the PQ group on day 7 due to the lack of infectious individuals.

Supplementary Table 6. Mean within-person percent change in hemoglobin from baseline across treatment groups.

Visit	Treatment group	% Change (range)	p-value*
Day 1	SP-AQ	-9·2 (-18·2, 4·4)	Ref
	SP-AQ +PQ	-7·2 (-15·9, 8·3)	0·33
	DP	-8·0 (-15·3, 14)	Ref
	DP +MB	-5·5 (-17·8, 3·8)	0·22
Day 2	SP-AQ	-10·3 (-20·4, 2·7)	Ref
	SP-AQ +PQ	-6·9 (-17·5, 7·1)	0·15
	DP	-6·6 (-17·6, 14·3)	Ref
	DP +MB	-5·2 (-20·7, 6·6)	0·56
Day 3	SP-AQ	-5·5 (-19·1, 8·0)	Ref
	SP-AQ +PQ	-4·4 (-17·1, 9·3)	0·62
	DP	-3·2 (-13·4, 25)	Ref
	DP +MB	-2·0 (-18·5, 14·7)	0·67
Day 7	SP-AQ	-4·2 (-16·9, 14·2)	Ref
	SP-AQ +PQ	-1·8 (-12·6, 17·4)	0·30
	DP	-6·4 (-18·1, 10·0)	Ref
	DP +MB	-6·8 (-24·4, 2·9)	0·87
Day 14	SP-AQ	-3·3 (-15·5, 11)	Ref
	SP-AQ +PQ	-0·3 (-13·7, 11·0)	0·22
	DP	-4·9 (-15·9, 9·0)	Ref
	DP +MB	-6·7 (-22·2, 4·5)	0·42
Day 28	SP-AQ	-4·4 (-40·0, 14·2)	Ref
	SP-AQ +PQ	1·7 (-4·1, 10·1)	0·056
	DP	-2·9 (-16·7, 17·1)	Ref
	DP +MB	-1·0 (-19·2, 9·8)	0·52
Day 42	SP-AQ	-0·6 (-12·0, 13·9)	Ref
	SP-AQ +PQ	3·2 (-15·1, 17·4)	0·19
	DP	-2·3 (-12·3, 12)	Ref
	DP +MB	-0·8 (-20·7, 15·7)	0·61

DP = dihydroartemisinin-piperaquine, MB = methylene blue, PQ = primaquine, Ref = reference, SP-AQ = sulphadoxine-pyrimethamine plus amodiaquine
* p-value represents two sample t-test used to test between-group differences.

SUPPLEMENTARY FIGURES

Supplementary Figure 1. Female to male (*Pfs25*:*PfMGET*) gametocyte sex ratio by group and visit. Gametocyte sex-ratios were calculated among samples with a total gametocyte density above 0·2 gametocyte/µL or 20 gametocytes in the 100µL blood sample. Note: DP = dihydroartemisinin-piperaquine, SLD PQ = single low-dose primaquine, SP-AQ = sulphadoxine-pyrimethamine amodiaquine

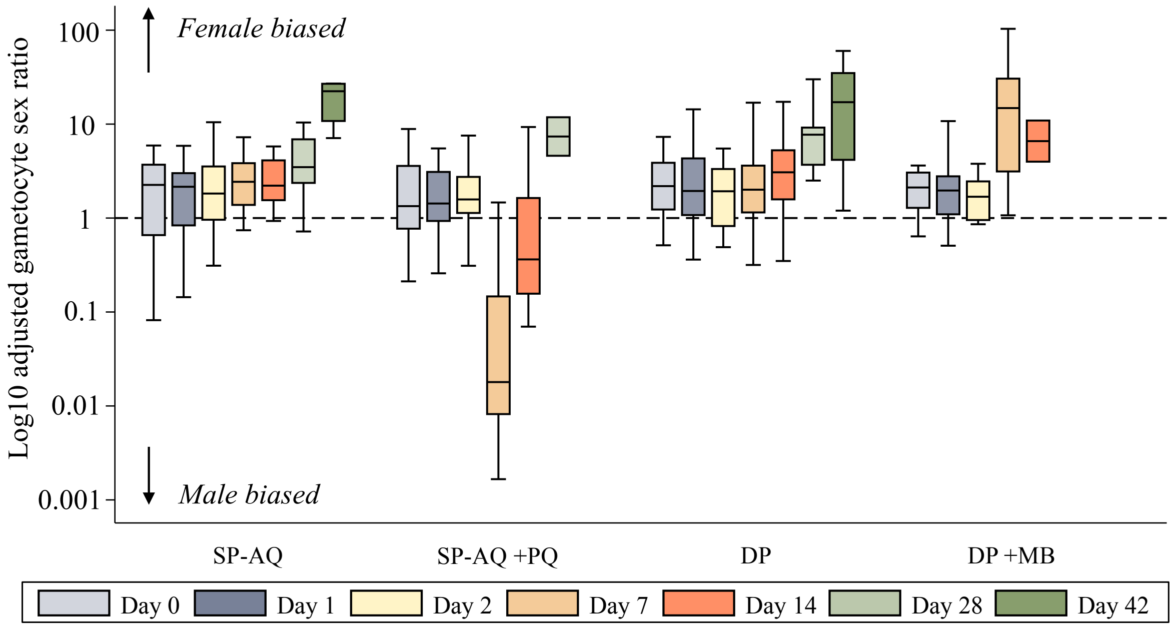

Supplementary Figure 2. Female (*Pfs25*) and male (*PfMGET*) gametocyte circulation times (days) by treatment group (mean [circle/box], 95% CI [whiskers]). P-values represent testing for between group differences. Note: DP = dihydroartemisinin-piperaquine, SLD PQ = single low-dose primaquine, SP-AQ = sulphadoxine-pyrimethamine amodiaquine.

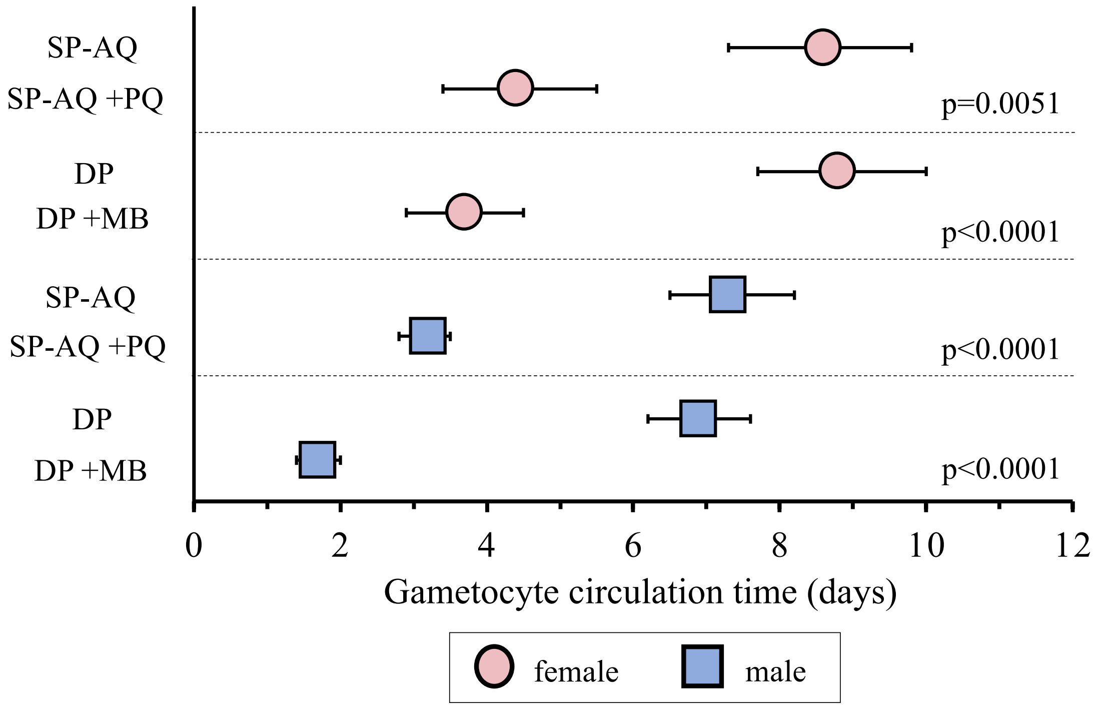

Supplementary Figure 3. The association between log10 adjusted female (*Pfs25*) and male (*PfMGET*) gametocyte densities and mosquito infection rates prior to treatment (A, C) and on day 7 post-treatment (B, D). SP-AQ and DP were combined to represent the non-gametocytocidal treatment groups. Note: DP = dihydroartemisinin-piperaquine, SLD PQ = single low-dose primaquine, SP-AQ = sulphadoxine-pyrimethamine amodiaquine.

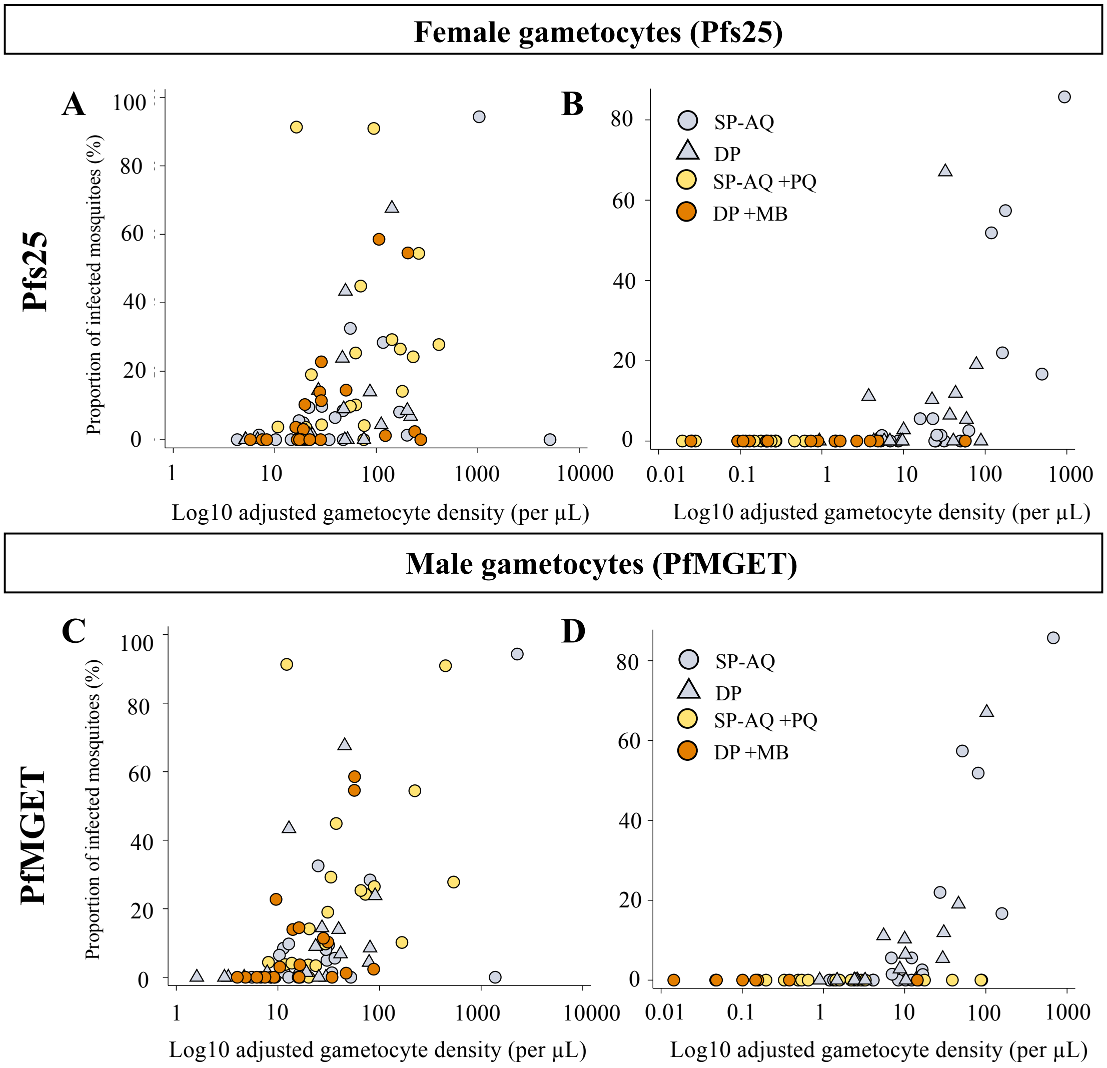

Supplementary Figure 4. Distribution (median [line], IQR [box], and range [whisker]) of haemoglobin values (g/dL) (A) and mean within-person percent change in haemoglobin (B) by group and visit. Note: DP = dihydroartemisinin-piperaquine, SLD PQ = single low-dose primaquine, SP-AQ = sulphadoxine-pyrimethamine amodiaquine.

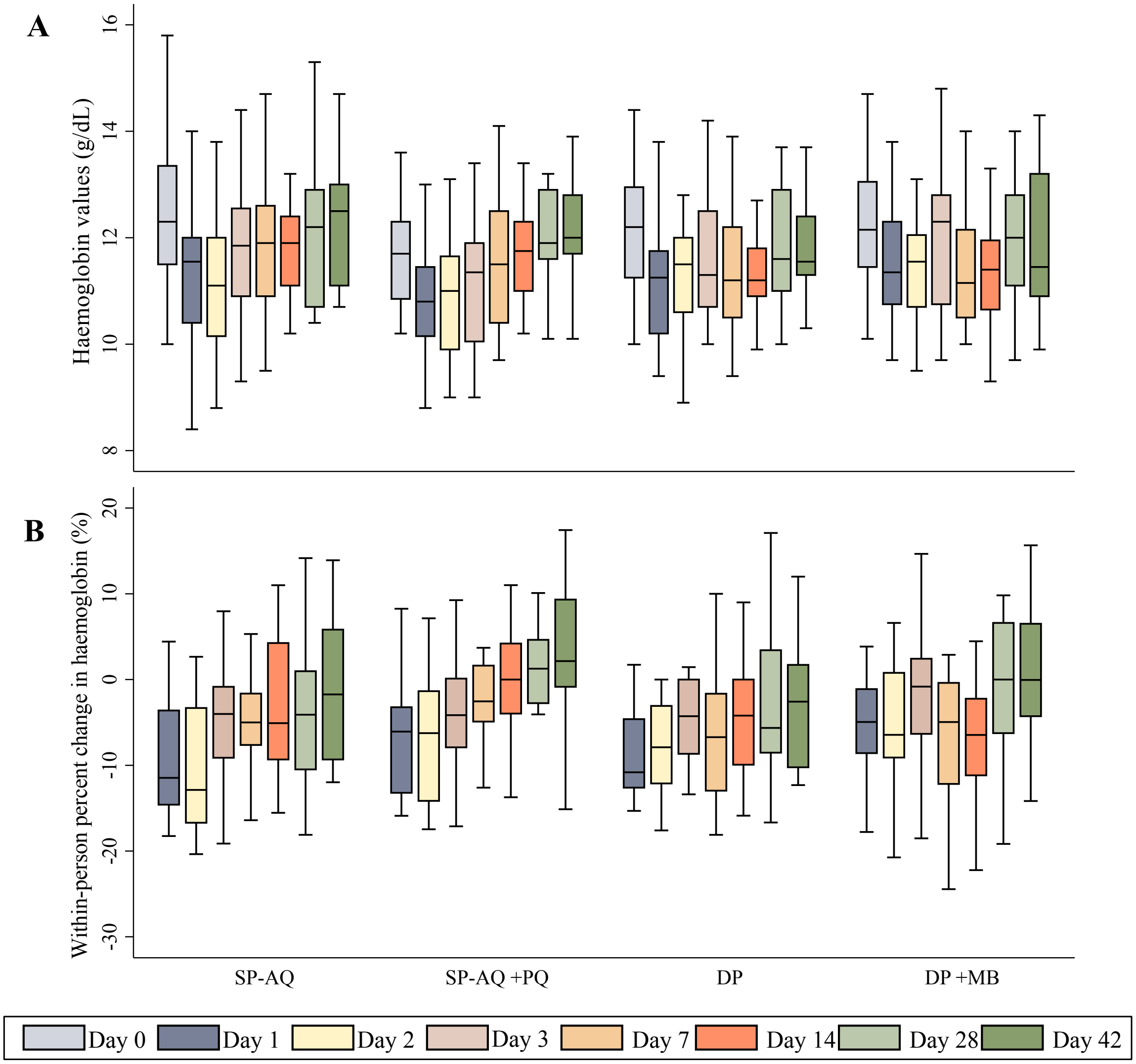

SUPPLEMENTARY MATERIALS: PROTOCOL

Phase 2, single-blind randomised controlled trial of the efficacy and safety of primaquine and methylene blue for preventing *Plasmodium falciparum* transmission in Mali

Principal Investigator

Prof Alassane Dicko
Faculty of Pharmacy and Faculty of Medicine and Dentistry, University of Sciences Techniques and Technologies of Bamako
Mali

Dr Roly Gosling
Global Health Group
University of California San Francisco
USA

Co-investigators Mali

Dr. Halimatou Diawara
Dr. Almahamoudou Mahamar
Dr. Harouna Soumare
Dr. Koualy Sanago
Dr. Djibrilla Issiaka
Dr. Amadou Barry
Dr. Issaka Sagara

Prof. Cheick Traore

Malaria Research and Training Centre
Faculty of Pharmacy and Faculty of Medicine and Dentistry, University of Sciences Techniques and Technologies of Bamako, Mali

Co-investigators International

Dr. Teun Bousema
University of Nijmegen
The Netherlands

Dr. Ingrid Chen
University of California San Francisco
USA

Dr. Joelle Brown
University of California San Francisco
USA

Dr. Jimee Hwang
Centers for Disease Control and Prevention
Atlanta
USA

Prof. Dr. Chris Drakeley
London School of Hygiene & Tropical Medicine
United Kingdom

Prof. Dr. Olaf Müller
University of Heidelberg
Germany

Planned start date: *November 2015*

Planned end date: *December 2016*

Table of Contents

Background 5
Statement of the problem 7
Drug safety 7
Objectives 10
Study site 10
Study design 11
Study population 11
Study drugs 11
Intervention 13
Outcome measures 13
Sample size calculation 13
Inclusion Criteria 14
Exclusion Criteria 14
Consent procedure 14
Study procedures 14
Randomization procedure 16
Blinding 17
Safety evaluation 17
Laboratory procedures 18
Data management and analysis 20
Ethical issues 20

List of abbreviations

AE: adverse event

AQ: amodiaquine

CRF: case reporting form

DNA: deoxyribonucleic acid

DP: dihydroartemisinin -piperaquine

DSMC: data and safety monitoring committee

EC: ethics committee

EDTA: Ethylenediaminetetraacetic acid

ELISA: enzyme-linked immunosorbent assay

GCP: good clinical practice

G6PD: glucose-6-phosphate dehydrogenase

MB: methylene blue

MDA: mass drug administration

MRCT: Malaria Research and Training Center

PCR: polymerase chain reaction

P. falciparum: *Plasmodium falciparum*

PQ: primaquine

QT-NASBA: quantitative nucleic acid sequence-based amplification

RT-qPCR: quantitative reverse transcriptase polymerase chain reaction

SAE: serious adverse event

SMC: seasonal malaria chemoprophylaxis

SP: sulphadoxine-pyrimethamine

WHO: World Health Organization

Summary
Title: Phase 2, single-blind randomised controlled trial of the efficacy and safety of primaquine and methylene blue for preventing Plasmodium falciparum transmission in Mali

Purpose: The purpose of this study is to determine the most efficacious transmission blocking drug regimen for seasonal malaria chemoprophylaxis in Mali. The primary outcome measure will be the proportion of mosquitoes infected pre and post-treatment, assessed through membrane feeding and measured by oocyst prevalence in mosquitoes dissected on day 7 post feed. Primary endpoint will be compared between the mean of the pretreatment infectivity (Day 0) and infectivity at 7 days post first dose.

Study design: This is a four arm, phase 2, individual-based randomized controlled trial. Participants will be randomized to receive:

- Sulphadoxine-pyrimethamine-amodiaquine, or;
- Sulphadoxine-pyrimethamine-amodiaquine + 0.25 mg/kg primaquine, or;
- Dihydroartemisinin-piperaquine, or;
- Dihydroartemisinin-piperaquine with methylene blue (15 mg/kg/day x 3 days)

Study Population: Afebrile G6PD-replete Malian men aged 5-50 years (inclusive), presenting with *Plasmodium falciparum* gametocytes

Study Size: This study will enroll 20 participants per dose group. If all dose groups are tested, this study will enroll approximately 80 participants.

Study visit and duration: Each participant will be followed for 42 days. Participants will be evaluated for infectivity using a membrane feeding assay at baseline, and on days 2 and 7 post drug administration. Participants will also be assessed for gametocytes, hemolysis, and adverse events at baseline, and on days 1, 2, 3, 7, 14, 28, and 42 after receiving the first dose of the study drug.

Primary objective**:**
1. Assess the change of infectivity of gametocytes from malaria infected patients following the administration of sulphadoxine-pyrimethamine-amodiaquine (SP-AQ), SP-AQ plus single low dose primaquine (SP-AQ-SLD PQ), dihydroartemisinin-piperaquine (DP) and DP plus methylene blue (DP-MB) in *P. falciparum* gametocyte carriers.

Secondary objectives
1. Assess the safety and tolerability of SP-AQ, SP-AQ-SLD PQ, DP and DP-MB through clinical assessments on followup, and the measurement of hemoglobin concentration and methemoglobin levels
2. Assess the effect of SP-AQ, SP-AQ-SLD PQ, DP and DP-MB on the clearance of male and female *P. falciparum* gametocytes
3. Determine the pharmacokinetics of SP-AQ with or without PQ, PQ and carboxyprimaquine when given with SP-AQ, DP with or without MB, and MB with DP, in malaria infected adults without G6PD deficiency
4. Explore cytochrome p450 polymorphisms in malaria infected adults without G6PD deficiency deficiency who received SP-AQ, SP-AQ-SLD PQ, DP or DP-MB.

Background

The success of malaria control programs in reducing malaria transmission has led to the call for elimination in many areas. As malaria transmission reduces with the scale up of traditional tools of malaria control such as indoor residual spraying and insecticide treated bed nets, the residual transmission often attributed to outdoor or early evening biting vectors becomes apparent. In addition, countries that have reached low endemicity have large malaria-free areas susceptible to re-introduction of the parasites through the movement of infected people. Thus, new tools that can target an alternative part of the parasite lifecycle would add to current malaria control and elimination strategies. Such a strategy could involve targeting the parasite in humans [1].

Such tools for targeting the parasite in humans include seasonal malaria chemoprophylaxis (SMC) where repeated therapeutic doses of antimalarials are given during the peak malaria transmission season to prevent morbidity in children. A meta-analysis of SMC studies in which a therapeutic course of sulphadoxine-pyrimethamine plus amodiaquine (SP-AQ) was given once per month to children under the age of 5 years during the peak malaria transmission season showed an 83% (95% CI: 72%, 89%) reduction in the incidence of clinical attacks of malaria and a similar reduction in the incidence of severe malaria [2]. In many African studies, SP-AQ has shown to be efficacious for the treatment of uncomplicated falciparum malaria and comparable to sulphadoxine-pyrimethamine plus artesunate (SP-AS) [3, 4]. SMC with SP-AQ is predicted to be both effective and cost-effective in many parts of Africa [5], is recommended by the WHO in highly seasonal transmission areas of the Sahel [6], and is implemented as policy over huge areas of the Sahel.

While SP-AQ is the main drug combination used for SMC, its use in eastern parts of Africa is hampered by widespread drug resistance against SP [4, 7, 8]. For this reason, the use of the artemisinin-based drug combination, dihydroartemisinin-piperaquine (DP), for SMC is gaining interest, and has been trialed in the Gambia [9], in Senegal [10], and in Uganda [11]. An additional potential benefit of DP is the long half-life of piperaquine, offering longer periods of protection [11, 12].

Regardless of whether SP-AQ or DP are used for SMC, both drug regimens share a common limitation: neither drug has efficacy against mature gametocytes of *P. falciparum* malaria, the parasite lifecycle stage responsible for ongoing transmission. When using SP-AQ, gametocytes persist for more than one month after successful treatment of blood stage infections [13]. The use of DP shortens gametocyte carriage times [14] and infectivity for at least one week [15, 16], and while a better transmission blocker than SP-AQ, it still leaves a window of infectivity that could be removed or shortened by the addition of a gametocytocidal drug. This raises the question of whether SMC drug regimens may be further improved through the addition of specific gametocytocidal drugs to SP-AQ or DP.

<u>*Potential gametocytocidal drugs for SMC*</u>

Primaquine (PQ) and methylene blue (MB) are two potent gametocytocidal compounds that are currently available for use in humans, both of which have shown to dramatically reduce gametocyte carriage when combined with non-artemisinin therapy or artemisinin-based combination therapy [17-19].

<u>Primaquine: efficacy</u>

Primaquine, an 8-aminoquinoline developed in the 1940s, has been licensed by the FDA as an antimalarial drug since 1952 for the radical treatment of *P. vivax* and *P. ovale* infections because of its unique ability to kill the dormant liver form of these parasites. For this indication primaquine is usually

prescribed over 14 days of treatment (7mg/kg total dose) administered once per day [20]. Besides the use in the radical cure of vivax or ovale malaria, primaquine also has a unique gametocytocidal action that no other registered antimalarial has. For this gametocytocidal indication, a single low dose (SLD) of primaquine is sufficient to clear the mature (stage V) gametocytes of *P. falciparum* parasites [21]. Thus, primaquine can prevent onward transmission of malaria when used in combination with an effective blood schizonticidal. The World Health Organization (WHO) recommends SLD primaquine (0.25 mg/kg), in conjunction with an artemisinin-based combination therapy (ACT), to prevent the onward transmission of malaria when treating falciparum malaria in areas of artemisinin drug resistance or malaria elimination. This recommendation is adopted as policy in 20 countries worldwide [22], and we recently showed that this dose is efficacious in preventing malaria infectivity when given with DP [16].

As SLD PQ + DP has already been studied for transmission blocking in mosquitoes [16], the impact of adding SLD PQ to SP-AQ remains to be established. To date, no studies have investigated the combination of SP-AQ and SLD PQ in Africa. One study was conducted in Colombia, where the impact of adding a single high dose of PQ (0.75 mg/kg) to SP-AQ on gametocytemia as assessed using microscopy [23]. In this study, gametocytemia was reduced, but not cleared, on days 4 and 8 of follow-up. The reasons for this are unclear; it is plausible that drug resistance against SP and AQ in Colombia [24] may cause the ongoing production of gametocytes. Regardless, there is a need to assess the impact of adding SLD PQ to SP-AQ in western Africa, where SP-AQ is used routinely, and drug resistance to SP-AQ is much less common than in many other regions of the world [25].

<u>Methylene blue: efficacy</u>

MB is a water-soluble dye that has been used for more than a century to treat malaria [26]. MB has high potency against *P. falciparum* gametocytes *in vitro* and *in vivo* [19, 27], and was recently identified as the most potent drug in clearing mature gametocytes *in vitro* [19]. Furthermore, MB preferentially clears mature male gametocytes over female ones [28], increasing its potential transmission blocking efficacy, as gametocytes are required to mate to propagate the malaria lifecycle [29].

Although MB is the oldest synthetic drug for the treatment of malaria [26], its widespread use was hampered by its colorative properties, turning the urine blue-green and – with very high doses – the sclera in the eyes a faint blue color [30]. Recent studies on MB are reported from Burkina Faso, and have explored doses of MB ranging from 4 mg/kg/day x 3 days [31], to 24 mg/kg/day (split into 1, 2, or 4 doses per day) x 3 days [32]. Studies have entailed the use of MB alone, with chloroquine, with artesunate, or with artesunate-amodiaquine, detailed in the following paragraph.

A study exploring the use of MB alone (13 mg/kg/day as 2 separate doses x 3, 5, or 7 days) in 60 participants found that MB acts slowly against asexual falciparum parasites, and appears to be able to clear parasites when given for at least 7 days, recommending that MB be administered with a schizontocidal partner drug [33]. Several trials have investigated the administration of MB with chloroquine, at 4 mg/kg/day x 3 days in young children (including those with G6PD-deficiency) [31], at 4.3 mg/kg/day x 3 days in 74 G6PD-deficient adult men [34], at 12, 18, or 24 mg/kg/day x 3 days in 435 children [32]. These studies found that MB in combination with chloroquine was not efficacious at low doses of 4 mg/kg/day x 3 day [31], that doses of 4.3 mg/kg/day x 3 days were safe in G6PD-deficient men [34], and that higher doses (12, 18, or 24 mg/kg/day x 3 days) showed limited efficacy against malaria, presumably due to widespread chloroquine resistance in Burkina Faso [32].

A second set of studies explored the use of MB with artesunate and/or amodiaquine. In one study, MB was administered at 20 mg/kg/day (as 2 separate doses) x 3 days with artesunate or amodiaquine to 180 children [18], showing that MB was effective in clearing gametocytes seen by microscopy by day 14 of followup. In another study published within a review of several of the above studies, MB was administered at 20 mg/kg/day x 7 days with artesunate, or at 20 mg/kg/day x 3 days with amodiaquine, analyzed as pooled safety data without report of efficacy [35]. Most recently, MB was administered at 15 mg/kg/day x 3 days with artesunate-amodiaquine in 221 children, showing that gametocyte prevalence on day 7 by microscopy and QT-NASBA was significantly lower with the addition of MB, and that asexual malaria clearance rates was similar in groups with and without MB [36].

This study showed that MB at 15 mg/kg/day x 3 days when administered with AS- AQ, an artemisinin-based drug and a partner drug with a long half-life,was efficacious in the clearance of both asexual parasitemia and gametocytes by day 7, as assessed by microscopy and QT-NASBA. However, several knowledge gaps for MB remain. The use of MB with DP has never been assessed, although DP is a promising partner drug regimen considering its similarities to AS-AQ, in containing an artemisinin-based component with a long-acting partner drug. The transmission-blocking properties of MB have never been confirmed through direct or membrane feeding assays, the latter of which is the gold standard for infectivity studies. There is a pressing need to assess the efficacy of MB through membrane feeding assays, as molecular methods that rely on the quantification of gametocytes are limited by their inability to differentiate between sterile or dead gametocytes from those that remain infective after treatment [29].

Statement of the problem

SMC entailing the use of monthly doses of SP-AQ throughout the rainy season is recommended by the WHO [6], and is implemented as policy over huge areas of the Sahel. SMC using DP offers potential benefits, including its implementation in east Africa, where drug resistance against SP is widespread. The potential impact of SMC using either SP-AQ or potentially DP could be increased through the addition of a gametocytocidal, transmission-blocking drug, but there is a lack of evidence on the effectiveness of adding a gametocytocidal drug, SLD PQ or MB, to DP or SP-AQ. To potentially optimize the drug regimen for SMC by increasing its impact on transmission, we propose to conduct a phase 2 randomized clinical trial. We will assess the impact of adding SLD PQ or MB to SP-AQ or DP on post-treatment infectivity in Mali, where SMC using SP-AQ is currently administered to all children under the age of 5 years in areas at risk of seasonal malaria [37]. We will investigate transmission-blocking effects in males age 5 and above for ethical reasons, as venous blood draws unsuitable for children under the age of 5 are required for infectivity assessments using the membrane feeding assay.

Drug safety: Primaquine, methylene blue, and glucose-6-phosphate dehydrogenase deficiency

Safety precautions must be taken in this study, as PQ and MB are both known to cause dose-dependent hemolysis among individuals who are deficient in glucose-6-phosphate dehydrogenase (G6PD), an enzyme involved in glucose metabolism prevalent in malarious geographies [38]. Hemolysis in G6PD-deficient (G6PDd) individuals is caused by oxidative stress induced by primaquine or methylene blue within red blood cells (RBCs), and can range in severity from compensated hemolysis, to mild acute hemolytic anemia (AHA), to severe AHA, with or without renal failure [21, 30, 39]. The extent of hemolysis depends on several factors including the dose of primaquine or methylene blue, the type of

G6PD deficiency, the functional G6PD assessment at the time of dosing, the concurrent use of other oxidant drugs, and whether the person is hemizygous (homozygous) or heterozygous for G6PD-deficiency [38].

To ensure the safety of all trial participants, we will only enroll G6PD-normal males in this study. There are two underlying reasons for this decision. The first is a biological reason; G6PD-deficiency is a sex-linked genetic trait as the G6PD gene is located on the X chromosome, putting hemizygous men and homozygous women at the greatest risk of hemolytic effects from enzyme deficiency. The second is a practical reason; hemizygous males and homozygous females tend to have the lowest levels of enzyme activity and are therefore the easiest to identify [38]. Identification of heterozygous females is complicated by X-chromosome mosaicism (or lyonization), as the use of common tests for G6PD activity, including the fluorescent spot test and/or Carestart test, are often unable to detect intermediate levels of G6PD activity common in heterozygous females [38]. As hemizygous males and heterozygous females are more common than homozygous females, it is practical to exclude all females in this study to ensure that heterozygous females do not get exposed to primaquine or methylene blue.

A further review of the safety of primaquine or methylene blue is detailed below. Although we do not anticipate any hemolytic adverse events in our patient population of G6PD-normal men, we will conduct safety precautions by conducting clinical assessments on follow-up, by offering care free of charge to all participants throughout the duration of follow-up, and by monitoring participant hemoglobin levels on each day of follow-up. Should any adverse events arise, hospital facilities will be available 24 hours a day, 7 days a week, and will be able to provide access to clean blood for transfusion according to standard precautions in Mali, and/or renal dialysis should they, in any circumstance, be needed.

1. Safety of 0.25 mg/kg dose of SLD PQ

Although we expect that a 0.25 mg/kg dose of SLD PQ is safe in both G6PD-normal and G6PD-deficient individuals, we will take extra safety precautions by excluding G6PD-deficient individuals in this study. Our basis for this expectation is the WHO recommendation to use this dose (0.25 mg/kg), without mandatory G6PD testing, in conjunction with an artemisinin-based combination therapy, to block *P. falciparum* transmission in areas threatened by artemisinin resistance or approaching malaria elimination [17]. The safety of SLD PQ is ascertained by its wide use over the past 60 years, where millions of individuals have received doses higher than 0.25 mg/kg without reported serious adverse events, although pharmacovigilance was noted to be weak [21]. Furthermore, the safety of SLD PQ in both G6PDd and G6PD-normal individuals has been confirmed in recent clinical trials. G6PDd individuals in Uganda who received up to a 0.4 mg/kg dose of SLD PQ did not experience statistically significant or clinically relevant reductions in hemoglobin concentrations [40], an observation consistent with those from the recent SAFEPRIM trial in Burkina Faso (unpublished findings, clinicaltrials.gov NCT02174900). Primaquine use is widespread throughout the world, and many countries have adopted the 0.25 mg/kg WHO recommendation for SLD PQ use as policy, without G6PD testing [30].

2. Safety of dose of MB: 15 mg/kg/day x 3 days

Several recent studies of MB, at or above 15 mg/kg/day for 3 days, provide evidence of its safety in G6PD-normal individuals over 6 months of age in Africa.

In 2013, a pooled analysis of 4 clinical trials investigated the safety and efficacy of methylene blue, where MB was given at 4 mg/kg/day, 12 mg/kg/day, or 24 mg/kg/day for 3 days with chloroquine, or 15

mg/kg/day or 20 mg/kg/day for 3 days with artesunate, or artesunate-amodiaquine without MB, in 1,005 children with uncomplicated falciparum malaria in Burkina Faso [35]. Of these 1,005 children, 20% (199) were G6PD-deficient, and the remaining 80% (806) were G6PD-normal. Out of 1005 children, there were two episodes of hemolysis leading to severe anemia (Hb ≤5 g/dL); a 21-month-old girl with heterogyzous G6PD deficiency received 4 mg/kg/day of MB x 3 days with a high initial parasitaemia of falciparum malaria (193,000 parasites/mL) and a 28-month-old boy with hemizygous G6PD deficiency received high doses of 24 mg/kg/day x 3 days of MB and developed severe anemia, which improved on iron supplementation [32]. No hemolytic serious adverse events were observed G6PD-normal individuals, in doses investigated that are equal to or similar to those we propose to investigate in this study. The authors noted that although children with hemi- or homozygous G6PD (A-) deficiency treated with 15 mg/kg per day of MB was associated with a statistically significant reduction in Hb values, the minimum value reached was of 8.5 g/dL and no clinical consequences were observed.

A subsequent study published in 2015 entailed the administration of MB (15 mg/kg/day for 3 days), with artesunate-amodiaquine (AS-AQ), to 221 children with uncomplicated falciparum malaria in Burkina Faso [36]. The MB used in this study was an improved pediatric formulation. There were no serious adverse events related to the study medication, and six cases of hemolysis, with the same number of hematological events in each group (three cases in the AS-AQ group, and three cases in the AS-AQ-MB group). Five individuals had a drop in Hb ≥ 2.5g/dL within 24 hours between day 0 and 1 (two in the AS-AQ group and three in the AS-AQ-MB group), and one individual had a Hb ≥ 2.5g/dL within 24 hours between days 2 and 3 (in the AS-AQ group). The G6PD status of individuals in this study was not reported. Hemoglobin concentrations were significantly lower in the AS-AQ-MB group than in the AS-AQ group on day 2 (P = 0.04) and day 7 (P = 0.005), and in both groups, hemoglobin values rapidly increased thereafter without therapeutic intervention, with hematological recovery being more rapid in the AS-AQ group than the AS-AQ-MB group. As G6PD status was not reported, it is unclear whether drops in Hb in this study were related to G6PD-deficiency. Regardless, no clinical consequences were observed, and hemoglobin values recovered without further intervention. In this study, vomiting was reported to be a side effect of MB; 25 children were excluded from the study because they vomited after drug administration, and 22/25 of these children received AS-AQ-MB [36].

It should be noted that an additional study was conducted in 2009, where methylene blue (20 mg/kg/day x 3 days) was given in conjunction with artesunate (4 mg/kg/day x 3 days) or amodiaquine (10 mg/kg/day x 3 days) in 180 children with uncomplicated falciparum malaria in Burkina Faso [18]. G6PD testing was not conducted for this study, nor were adverse events reported.

Taken together, the evidence presented above, from six clinical trials including approximately 1,400 individuals, suggests that a 15 mg/kg/day dose of MB for 3 consecutive days in African individuals may lead to mild hemolysis that is similar to the use of SLD-PQ. The resulting hemolysis and mild anemia resulting in no clinical symptoms self corrects rapidly, without intervention, as was previously reported for SLD-PQ [41]. As stated above, although we do not expect severe hemolytic adverse events from this dose of MB, precautions will be taken to ensure that if clinically significant hemolysis does occur, that patient(s) will have immediate access to care in both a local health center and regional hospital. As the nadir in hemoglobin concentrations after MB and SLD PQ occur within the first week after drug administration, the study participants will be intensively monitored during this period in the current study.

Objectives

Primary specific objective

1. Assess the change of infectivity of gametocytes from malaria infected patients following the administration of sulphadoxine-pyrimethamine-amodiaquine (SP-AQ), SP-AQ plus single low dose primaquine (SP-AQ-SLD PQ), dihydroartemisinin-piperaquine (DP) and DP plus methylene blue (DP-MB) in *P. falciparum* gametocyte carriers.

Secondary specific objectives

1. Assess the safety and tolerability of SP-AQ, SP-AQ-SLD PQ, DP and DP-MB through clinical assessments on followup, and the measurement of hemoglobin concentration and methemoglobin levels
2. Assess the effect of SP-AQ, SP-AQ-SLD PQ, DP and DP-MB on the clearance of male and female *P. falciparum* gametocytes
3. Determine the pharmacokinetics of SP-AQ with or without PQ, PQ and carboxyprimaquine when given with SP-AQ, DP with or without MB, and MB with DP, in malaria infected adults without G6PD deficiency
4. Explore cytochrome p450 polymorphisms in malaria infected individuals without G6PD deficiency deficiency who received SP-AQ, SP-AQ-SLD PQ, DP or DP-MB.

Study site

The Malaria Research and Training Centre (MRTC) in Bamako, Mali will use the field site of Ouelessebougou for recruitment. The MRTC has the unique position of being one of very few sites in Africa that can study malaria transmission endpoints. Between 2013 and 2014, the MRTC successfully carried out a very similar trial at this site with great success, in collaboration with the University Nijmegen, the Netherlands, the Mahidol Oxford Research Unit, Thailand, and the University of California San Francisco. In the 2013-2014 study, the MRTC demonstrated the efficacy of SLD PQ of doses of 0.125mg/kg or greater in combination with dihydroartemisinin-piperaquine for blocking *P falciparum* transmission safely in G6PD normal Malian males from the age of 5-50 years [16]. MRTC has already established all the assays required for the study described in this protocol. This collaboration is now carrying out a study to test the safety of doses not to exceed 0.75 mg/kg of primaquine in G6PDd Malian adult males. The results should be available later this year.

The MRTC is experienced in conducting Good Clinical Practice (GCP) compliant clinical trials including phase 2 malaria vaccine and drug studies. The site is in preparation for future evaluation of transmission blocking vaccines. Long term collaborations with the National Institutes of Health, USA, the University of Nijmegen, The Netherlands and other American and European institutions, have built capacity in molecular gametocyte measurement and mosquito infectivity. Ouelessebougou village has been conducting ongoing clinical research since 2006 and is endemic for malaria with marked seasonalityand a high burden of malaria in both children and adults populations. In recent years the prevalence of *P. falciparum* malaria in children under 5 years of age has ranged between 14% and 26% during the transmission season. The frequency of G6PD deficiency is in the range of 10 to 15%.

Study design
This is a four arm, individual-based randomized clinical trial. The study will be conducted according to the following steps for each individual:

1. Individuals aged ≥ 5 years with microscopically detectable *P. falciparum* gametocytes will be recruited from the community. After written informed consent, individuals will be enrolled and treated with AQ-SP, AQ-SP-SLD PQ, DP or DP-MB.
2. Study participants are followed up for 42 days. The safety of study participants, specifically signs of hemolysis will be closely followed.
3. Blood samples will be collected to measure infectivity, specifically:
    a. Three venous blood samples from study participants will be used in membrane feeding experiments
    b. Frozen blood samples will be analyzed using molecular methods off site, to determine the effect of study drugs on male and female gametocytes.

Study population
The study population will be derived from individuals aged ≥5 years with asymptomatic *P. falciparum* malaria who agree to be screened for malaria infection at the clinic. Those who are harboring microscopically detectable concentrations of *P. falciparum* gametocytes at densities >30 gametocytes/µL will be eligible. This lower threshold density for *P. falciparum* gametocytaemia is chosen to maximize pre-treatment transmission potential [42] and therefore the efficiency of pre- and post-treatment comparisons of infectivity. Each study arm will have a minimum of 20 study participants. The maximum weight of individuals is 80kg, to allow adequate dosing of DP (for which no dose recommendations are available for individuals >100kg) and for MB (where dosing has not previously exceeded 800mg/day)

Study drugs
The study drugs to be tested will be:
1. Amodiaquine-sulphadoxine-pyrimethamine (SP-AQ)

Participants in this arm will receive sulphadoxine pyrimethamine (Fansidar; Roche) as a single dose per manufacturer's dosing instructions as described below. Each Fansidar tablet is scored, containing 500 mg sulfadoxine and 25 mg pyrimethamine. Doses are administered by weight: 11-20 kg, 1 tablet; 21-30 kg, 1 ½ tablets; 31-45 kg, 2 tablets; >45 kg, 3 tablets. Fansidar will be given in combination with amodiaquine (Guilin Pharmaceutical) once daily for 3 days, per the following dosing instructions for 150 mg tablets:

Weight (kg)	Amodiaquine Number of Tablets					
	Tablets, 150-153 mg of base			Tablets, 200 mg of base		
	Day 1	Day 2	Day 3	Day 1	Day 2	Day 3
15-18	1.5	1	1	1	1	1
19-24	1.5	1.5	1.5	1.5	1	1
25-35	2.5	2.5	2	2	2	1.5
36-50	3	3	3	3	2	2
50+	4	4	3	3	3	3

2. AQ-SP plus single dose primaquine (SP-AQ-SLD PQ)

Participants in this arm will receive SP-AQ as above in combination with PQ (Sanofi) at a single low dose of 0.25mg/kg as is currently recommended by the World Health Organization. The single dose of PQ will be given on d0 together with SP-AQ, administered in an aqueous solution, according to a standard operating procedure (SOP) provided by the manufacturer [43]. Primaquine is absorbed rapidly and peak concentrations are reached in approximately 2 hours. It has a half-life of 6 hours and is metabolized in the liver with a large volume of distribution [44]. The metabolically inert principle metabolite (carboxy-PQ) reaches peak concentrations within 6 hours of administration [44]. The active metabolite has not yet been identified. The kinetics of PQ are affected by malaria (acute infection reduces oral clearance of PQ) [45], by food (increased PQ bio-availability) [46], or by other antimalarials (quinine induces a higher area under the curve (AUC) of the carboxy metabolite [47]).

3. Dihydroartemisinin-Piperaquine (DP)

Participants in this arm will be treated with standard doses of DP. Tablets containing 40 mg dihydroartemisinin/320 mg piperaquine tablets (Eurartesim, Sigma Tau) will be administered per manufacturer guidelines shown below [48]:

Body weight (kg)	Daily dose (mg)<br>(to be given 1x/day for 3 days)		Tablet strength and number of tablets per dose
	Piperaquine	DHA	
5 to <7	80	10	½ x 160mg / 20mg tablet
7 to <13	160	20	1 x 160mg / 20mg tablet
13 to <24	320	40	1 x 320mg / 40mg tablet
24 to <36	640	80	2 x 320mg / 40mg tablet
36 to <75	960	120	3 x 320mg / 40mg tablet
75 to 80	1,280	160	4 x 320mg / 40mg tablet
>80	Not eligible		

4. DP plus methylene blue (DP-MB)

Study participants in this arm will receive DP as described above combined with once-daily MB for 3 days, at 15 mg/kg/day (45 mg/kg total over 3 days). MB will be given as minitablets in prepackaged sachets according to weight groups (6.0–8.9 kg, 100 mg MB; 9.0–12.9 kg, 150 mg MB; 13.0–16.9 kg, 200 mg MB [36]; 17.0-19.9 kg, 250 mg MB; 20.0 – 23.0 kg, 300 mg MB; 23.1 – 29.0 kg, 400 mg MB; 29.1 – 36.0 kg, 500 mg MB; 36.1 – 43.0 kg, 600 mg MB; 43.1 – 50.0 kg, 700 mg MB; 50.1 – 80.0 kg, 800 mg MB. The MB minitablets were developed at the Düsseldorf University in Germany, and were produced by the Pharbil Waltrop company in Germany under good manufacturing practice conditions [36].

Intervention

The intervention will be a four arm randomized control trial. Eligible participants will be randomized to one of the following treatments:

AQ-SP, AQ-SP-SLD PQ, DP or DP-MB.

Outcome measure

The primary outcome measure will be the absolute change in the proportion of mosquitoes infected pre and post-treatment, assessed through membrane feeding and measured by oocyst prevalence in mosquitoes dissected on day 7 post feed. The primary endpoint will be a comparison between the mean of the pretreatment infectivity (Day 0) and infectivity at 7 days post first dose. Our study is not designed to compare the transmission-blocking effects between treatment arms.

Secondary outcome measures include: presence of oocysts in mosquitoes at other time points, gametocyte prevalence, density, and sex ratio, determined microscopically and by molecular methods, asexual parasite prevalence and density, safety assessment including hemoglobin measurement, methemoglobin measurement, and clinical review (including additional signs of hemolysis) on active and passive follow-up, PQ, MB, SP, AQ, and DP pharmacokinetics, and the identification of cytochrome P450 (CYP) single nucleotide polymorphisms through genotyping.

Table 2. Outcome measures

Outcome measure	Day 0	Day 1	Day 2	Day 3	Day 7	Day 14	Day 28	Day 42
Mosquito infectivity: Prevalence of oocyst infected mosquitoes	*		*		*			
Gametocyte density	*	*	*	*	*	*	*	*
Gametocyte sex-ratio	*	*	*	*	*	*	*	*
Parasite stage composition	*	*	*	*	*	*	*	*
Hemoglobin	*	*	*	*	*	*	*	*
Safety assessment: signs of hemolysis	*	*	*	*	*	*	*	*
Pharmacokinetics of study drugs[†]	*	*						
CYP 2D6 sampling	*							

[†]only in adult participants ≥ 18 years of age

Sample size calculation

Sample size estimation was based on efficacy for single dose primaquine to provide a 95% or greater reduction in infectivity at 7 days post initiation of treatment compared to pretreatment [16]. SLD PQ was used to derive sample sizes because measures of infectivity using membrane feeding, as will be done in this study, have only been conducted using SLD PQ and not MB. For all MB studies reported to date, only gametocyte density has been used as surrogate indicator for infectivity. Gametocyte density measurements are less accurate than membrane feeding assays, the gold standard, because gametocytes may become non-infective or may die, but may still be captured in gametocyte density measurements [16, 49], causing gametocyte measurements to underestimate drug effects on infectivity. Our previous

study confirmed this by showing high levels of persisting gametocytes after SLD PQ that contrasted with a complete cessation of infectivity beyond day 2 [16].

In this study, a membrane feeding assay will be used, and infectivity will be assessed by oocyst prevalence in mosquitoes dissected at day 7 after feeding. In a previous study using a membrane feeding assay at this study site, 79% of individuals infected at least one mosquito pretreatment, and among those who infected at least one mosquito, an average of 21% of mosquitoes had oocysts. In that study all intervention arms with primaquine had reached zero infectivity by day 7 and control had declined by only 66%. 20 participants per group, we will have 80% power to detect a 95% or greater reduction in the number of mosquitoes with oocysts after treatment as significant at the 0.05 level. Our sample size is insufficient to compare the transmission reducing effects of treatment arms and analyses will focus on the change in infectivity within treatment arms.

Inclusion Criteria:

- Age ≥ 5 years and ≤ 50 years
- Male gender
- G6PD-normal defined by Carestart rapid diagnostic test or the OSMMR2000 G6PD qualitative test
- Absence of symptomatic falciparum malaria, defined by fever on enrolment
- Presence of *P. falciparum* gametocytes on thick blood film at a density >30 gametocytes/µL (i.e. ≥2 gametocytes recorded in the thick film against 500 white blood cells)
- Absence of other non-*P. falciparum* species on blood film
- No allergies to study drugs
- No use of antimalarial drugs over the past 7 days (as reported by the participant)
- Hemoglobin ≥ 10 g/dL
- Individuals weighing < 80 kg
- No evidence of severe or chronic disease
- Written, informed consent

Exclusion criteria

- Age < 5 years or > 50 years
- Female gender
- Blood thick film negative for sexual stages of malaria
- Detection of a non-*P. falciparum* species by microscopy
- Previous reaction to study drugs / known allergy to study drugs
- Signs of severe malaria, including hyperparasitemia (defined as asexual parasitemia > 100,000 parasites / µL)
- Signs of acute or chronic illness, including hepatitis
- The use of other medication (with the exception of paracetamol and/or aspirin)
- Consent not given

Consent procedure

Consenting procedures will vary based on age of the potential participant. Participants aged 18 years and above will provide informed consent. For participants under 18 years of age we will seek parental consent. In addition to parental consent, assent will be sought for children aged 12-17 years. The Ethics committee in Mali does not require that participants under the age of 12 provide assent. The written, informed consent procedure will be conducted in French or in a local language understood by subject as described in the information and consent form. If the participant is unable to read or write, a fingerprint will be used as an official signature.

Study procedures

Participants who agree to participate and provide written, informed consent will be assessed for the presence of sexual blood stage parasite with a thick blood film and rapid diagnostic test and G6PD activity (Patients who are willing to be screened will undergo a G6PD test (Carestart™ 3 G6PD, Access Bio, USA or OSMMR-2000 G6PD test, R&D Diagnostics Ltd ®, Greece) from a finger prick sample. Those with sexual stages seen on the thick film and normal G6PD activity will be eligible for enrolment into the study. Individuals with a positive malaria slide with asexual stages only will not be enrolled in the study, but will be treated with artemether + lumefantrine, the standard treatment for uncomplicated malaria as per the Ministry of Health policy in Mali.

Eligible patients will then be randomized (see below under randomization procedure) to one of the treatment groups, and drugs will be administered. In case of vomiting within 30 minutes after drug intake, the drugs will be re-administered once [36].

Adult participants (age $\geq$ 18 years) will be requested to stay at the research facility for up to 8 hours so as to enable frequent sampling for infectivity and for pharmacokinetics. Adult participants will be given the reasons for frequent sampling and explained the risks associated with the procedure. Pharmacokinetic samples will not be taken on participants under the age of 18 years. The participants will be compensated for any travel costs and for work loss income. Participants will be informed that in addition to the testing for malaria (and for adults only, drug levels), they will be tested for their ability to metabolize drugs (cytochrome testing) and they will be tested for genotypic risk factors for hemolysis, such as G6PD deficiency. Participants will be followed for 42 days as described in Table 3 (below). In adult participants, blood samples will be taken on 13 occasions in up to 8 visits (total blood volume approximately 30ml) and are shown in the sampling framework below. Among participants under the age of 18 years, blood samples will be taken on 8 occasions in 8 visits (total blood volume of approximately 27 ml).

The sampling framework is below. Single venous draws or finger pricks will be used. Pharmacokinetic samples will only be collected in participants aged 18 years and older.

Table 3. Sampling frame for human participants

Time of sampling (h = hour, d = day)	0 h	1 h	2 h	4 h	6 h	8 h	24 h/ d 1	D 2	D 3	D 7	D 14	D 28	D 42
**Blood sampling**													
Type of sampling[+]	V	C	C	C	C	C	V	V	C	V	V	V	V
Mosquito infectivity (3 mL)	*							*		*			
Gametocyte density, sex ratio and parasite stage composition (2 ml*)	*						*	*		*	*	*	*
Blood smear for asexual parasite and gametocyte density (0.1ml)	*						*	*	*	*	*	*	*
Hemoglobin (0.1 mL)	*						*	*	*	*	*	*	*
G6PD activity (0.5ml)	*												
Pharmacokinetics (0.5ml)†		*	*	*	*	*	*						
CYP and G6PD genotyping (1.0 mL)	*												
**Total Volume of blood sampled adults† (mL)**	**7.2**	**0.5**	**0.5**	**0.5**	**0.5**	**0.5**	**2.5**	**5.2**	**0.2**	**5.2**	**2.2**	**2.2**	**2.2**
**Total Volume of blood sampled age < 18 years (mL)**	**7.2**	**0**	**0**	**0**	**0**	**0**	**2.2**	**5.2**	**0.2**	**5.2**	**2.2**	**2.2**	**2.2**
**Non-invasive sampling**													
Noninvasive methemoglobin measurement	*						*	*	*	*	*	*	*

[+] V denotes venous blood sampling and C denotes capillary sampling from a finger prick.
[†] only in adult participants ≥18 years of age
* Except on day 1, when 1.7 mL will be drawn

Blood volume justification

In the table below (table 4), the justification of the blood volumes is presented.

Table 4. Sampling volumes and justification for human participants

Day	Assay (volume)	Pricking method	Total volume
0 (0 h)	Mosquito feeding assays: single heparin tube 3mL G6PD enzyme activity test (0.5mL required); magnetic enrichment and depletion of gametocytes (2mL required); Hb (0.1mL	1 venous blood sample, two tubes used	7.2mL (3mL heparin tube; 3mL EDTA tube)

	required), slide (0.1mL required): G6PD and CYP2D6 genotyping (1mL) single EDTA tube 3mL		
0 (1h) only in adults	PQ, MB, SP, AQ, and DP pharmacokinetics (0.5mL EDTA)	1 finger prick, 1 microtainer tube	0.5 mL (0.5mL EDTA microtainer)
0 (2h) only in adults	PQ, MB, SP, AQ, and DP pharmacokinetics (0.5mL required)	1 finger prick, 1 microtainer tube	0.5 mL (0.5mL EDTA microtainer)
0 (4h) only in adults	PQ, MB, SP, AQ, and DP pharmacokinetics (0.5mL required)	1 finger prick, 1 microtainer tube	0.5 mL (0.5mL EDTA microtainer)
0 (6h) only in adults	PQ, MB, SP, AQ, and DP pharmacokinetics (0.5mL required)	1 finger prick, 1 microtainer tube	0.5 mL (0.5mL EDTA microtainer)
0 (8h) only in adults	PQ, MB, SP, AQ, and DP pharmacokinetics (0.5mL required)	1 finger prick, 1 microtainer tube	0.5 mL (0.5mL EDTA microtainer)
1 (24h)	PQ, MB, SP, AQ, and DP pharmacokinetics (0.5mL required)<br>Magnetic enrichment and depletion of gametocytes (1.7mL required); Hb (0.1mL required), slide (0.1mL required): single EDTA tube 2mL	1 venous blood sample, 1 tube	2.5 mL (2mL EDTA tube) in adults<br>2.2mL (2mL EDTA tube) in children
2	Mosquito feeding assays: single heparin tube 3mL<br>Magnetic enrichment and depletion of gametocytes (2mL required); Hb (0.1mL required), slide (0.1mL required): single EDTA tube 2mL	1 venous pricking event, two tubes used	5.2mL (3mL heparin tube; 2mL EDTA tube)
3	Hb (0.1mL required), slide (0.1mL required):	1 finger prick, 1 microtainer tube	0.2 mL (0.5mL EDTA microtainer)

7	Mosquito feeding assays: single heparin tube 3mL Magnetic enrichment and depletion of gametocytes (2mL required); Hb (0.1mL required), slide (0.1mL required): single EDTA tube 2mL	1 venous pricking event, two tubes used	5.2mL (3mL heparin tube; 2mL EDTA tube)
14	Magnetic enrichment and depletion of gametocytes (2mL required); Hb (0.1mL required), slide (0.1mL required): single EDTA tube 2mL	1 venous pricking event, one tube used	2.2mL (2mL EDTA tube)
28	Magnetic enrichment and depletion of gametocytes (2mL required); Hb (0.1mL required), slide (0.1mL required): single EDTA tube 2mL	1 venous pricking event, one tube used	2.2mL (2mL EDTA tube)
42	Magnetic enrichment and depletion of gametocytes (2mL required); Hb (0.1mL required), slide (0.1mL required): single EDTA tube 2mL	1 venous pricking event, one tube used	2.2mL (2mL EDTA tube)

Risks of the trial will be clearly stated including the risk of hemolysis, which is thought to be low in G6PD normal people. All participants will have 24 hour access to a research clinician. The study site has a research clinic with an inpatient unit. Study physicians are available 24 hours a day seven days a week. The health facility has provision for giving safe blood transfusions. The tertiary care unit is in Bamako, approximately 80 km from Ouelessebougou, which will act as a backup facility should serious adverse events such as acute renal failure occur, as dialysis facilities are available there. There is a secondary hospital in Ouelessebougou with an ambulance available.

Randomization procedure

Eligible participants will be assigned the next sequentially-numbered study ID number based on a pre-printed Study ID List created by a study investigator based at UCSF by individual computer generated randomization to arms 1-4 in a ratio 1:1:1:1. The study pharmacist in Mali will open the corresponding sealed, opaque envelope and provide the intervention to study participants.

Blinding

The study is unblinded for patient, study pharmacist, and treating physician but is blind for the staff involved with assessing all laboratory outcomes of the study conducted off-site and the analysis.

Safety evaluation

The major safety endpoint is hemolysis. For this reason, hemoglobin will be measured before treatment, at days 1, 2, 3, 7, 14, 28, and 42. Previous studies have shown that highest hemoglobin fall related to drug induced hemolysis can be detected within 7 days after treatment. In addition at 24 hours, and

subsequent days of follow up, a questionnaire assessing adverse events (AE) will be carried out. All participants will have access to contact study medical staff 24 hours a day and all medical facilities can give safe blood transfusions.

All adverse events will be recorded on individual CRF with the following information:

- the severity grade (mild, moderate, severe)
- its relationship to the study drug(s) (related/not related)
- its duration (start and end dates or if continuing at final exam)
- actions taken
- outcome
- whether it constitutes a serious adverse event (SAE).

An adverse event will be defined as the appearance or worsening of any undesirable sign, symptom, or medical condition occurring after starting the study drug even if the event is not considered to be related to study drug.

A Data and Safety Monitoring Committee (DSMC) will be established and convened before the onset of the trial to agree stopping points for each study arm with regards to safety and efficacy. The DSMC will be informed of any serious adverse event within 48 hours of the event being notified to study personnel. An interim analysis will be carried out after the first 40 subjects have been enrolled to examine safety endpoints. Additional interim analysis may be requested and done by the DSMC if they feel useful in advising the study. Throughout the study, DSMC will have access to unblinded data.

Treatment failure criteria
In case a patient develops recurrence of symptomatic malaria (denoted as fever and the presence of asexual parasitemia denoted by microscopy) at any time during followup, he will be treated with a full course of artemether-lumefantrine (Coartem, Novartis), the first-line antimalarial in Mali [50] . Each tablet of Coartem consists of 20 mg artemether and 120 mg lumefantrine, where a total of 6 doses are to be given over 3 days according to bodyweight per manufacturer's dosing instructions [51]:

5 to <15 kg	1 tablet
15 to <25 kg	2 tablets
25 to <35 kg	3 tablets
35 kg and over	4 tablets

Laboratory procedures

A. Efficacy and Infectivity measurements

Blood film

Thick and/or thin blood films for parasite counts will be obtained and examined at screening to confirm *P. falciparum* monoinfection. Giemsa-stained thick and/or thin blood films will be examined at a magnification of 100×. Smear will be considered negative if no parasites are seen after examining 100 high powered fields.

Mosquito infectivity assay

For each assessment of infectivity 3 ml of heparinized blood will be drawn from the study participant and stored at 37°C and transported to the insectary. At the insectary, using standard procedures, ~90 *A. gambiae* will be fed on the subjects' blood for 15-20 minutes (this figure is based on a previous study where 61 fed mosquitoes allowed for an average of 50 mosquitoes surviving until day 7 after the feeding experiment and being available for dissection and examination for oocysts). All of these mosquitoes will be dissected on the 7th day after the feeding assay for prevalence of mosquitoes with oocysts and quantification of oocysts. Infected guts will be stored for later PCR confirmation of oocysts.

Gametocyte and asexual stage density measurement

Blood slides stained with Giemsa will be double read over 500 fields for quantification of gametocytes and asexual stages. EDTA samples of blood (1 mL) will be tested for molecular quantification of gametocytes and asexual parasites. Parasite DNA and RNA will be extracted from whole blood samples in EDTA tubes and tested using quantitative real-time polymerase chain reaction (qPCR) with a detection limit of 0.02-0.1 parasites/µL of blood and highly precise parasite quantification [52]. Quantification of gametocytes will be based on Pfs25 mRNA detection and quantification by reverse transcriptase-PCR and quantitative nucleic acid sequence based amplification (QT-NASBA) [53].

Detailed analysis of Plasmodium stage composition will be done by quantitative RT-PCR where mRNA up-regulated in mature and immature gametocytes is quantified in a multiplex assay [54]. In addition, multiplicity of infection will be determined by merozoite surface protein (MSP) genotyping [55] and barcoding approaches [56] in 500µL whole blood samples and 1mL blood samples after gametocyte enrichment using magnetic columns [57, 58]. We have adapted this technique to prevent gametocyte activation and generate three populations of infected cells for each sample: one containing the actual distribution of lifecycle stages in host blood (usually >95% asexual ring stage parasites) and two populations after magnetic fractionation, one enriched in gametocytes and the other depleted in gametocytes).

All samples will be analyzed by qRT-PCR stage composition assay, molecular gametocyte sex determination and assessment of complexity of infection. A novel multiplex qRT-PCR stage composition assay with 5 markers will quantify asexual rings (R; PFE0065w), trophozoites/schizonts (T; PF10_0020), immature gametocyte (IG; PF14_0748), mature gametocytes (MG; PF14_0367) and the constitutive parasite marker (U; PF11_0209). Complexity of infections in the different fractions is determined by genotyping the polymorphic parasite gene MSP-2 with fragment sizing by capillary electrophoresis [59]. A newly developed gametocyte sex-specific qRT-PCR that is based on recent sex-partitioning of the *P. falciparum* proteome [60] and indicated Pfs25 mRNA as a suitable female marker

(at least 2.5 fold more abundantly expressed in female gametocytes) and P230p as a suitable male marker (PF3D7_0208900, at least 8-fold more abundantly expressed in male gametocytes). The ratio of Pfs25/P230p copy numbers is used as indicator of sex-ratio and extrapolated to densities/μL using pure in vitro cultured male and female gametocytes.

B. Safety

Hemoglobin concentrations

Haemoglobin concentrations will be also measured regularly throughout the study using the HemoCue system (Hemocue AB, Angelholm, Sweden).

Measurement of methemoglobin

Methemoglobin will be measured with a Masimo Rad-57 Pulse Oximeter, a noninvasive device that monitors for methemoglobin levels according to its light absorptive properties through a fingertip probe. Multiple primaquine safety studies are using this device to measure methemoglobin, the goal of which is to determine if methemoglobin can be used as a biomarker for hemolysis.

C. Additional measurements

G6PD testing

On screening, a 0.5 mL blood sample will be collected from each subject, to conduct G6PD testing as described below.

i. Qualitative G6PD testing

All subjects will have a qualitative assessment of G6PD activity measured from a drop of blood (approx. 0.05 mL), using a Carestart 3 point of care diagnostic test or a the OSMMR2000 G6PD test. The Carestart 3 test is a colormetric test that uses a tetrazolium compound to produce a purple readout for G6PD normal individuals or a colorless readout for G6PDd individuals. The test has shown to have a high sensitivity for levels of G6PDd that are below 2 units / g hemoglobin (<19% enzyme activity: severe to mild deficiency), and to detect the majority of tested samples with G6PDd at between 2 to 4 units / g hemoglobin (exact numbers tested not specified, corresponds to 19-38% enzyme activity, mild deficiency) [61]. Qualitative G6PD testing may also be carried out using the OSMMR2000-DG-6-PD, which also uses a tetrazolium salt to produce a colored readout for G6PD-normal individuals, or a colorless readout for G6PDd individuals.

ii. (Semi-)Quantitative G6PD testing

Following qualitative assessment of G6PD activity, the remainder of the blood sample for G6PD testing (approx. 0.4 mL) will be used for either a) a quantitative assessment of G6PD activity measured using a spectrophotometric validated assay or b) semi-quantitative G6PD testing using the OSMMR2000 G6PD test. Fresh whole blood is needed for quantitative spectrophotometry which can be conducted on frozen samples in Bamako, while either fresh whole blood or filter paper samples are appropriate for analysis using the OSMMR-2000-AN/50 enzymatic colormetric assay.

Pharmacokinetic measurement

In adult participants, 0.5 ml whole blood will be collected in appropriate collection tubes containing ETDA as the anticoagulant. The tube should be at room temperature (18°-25° C) prior to use. The

sample may be stored at ambient temperature for up to 2 hours before processing, after which the blood sample will be transferred to a screw cap cryovial (Nalgene No.: 5000 0012). The whole blood samples will be frozen at -80°C or below. Drug concentrations will be determined using LCMS (liquid chromatography and mass spectrometry) measured by the pharmacology department of the Mahidol Oxford University Research Unit in Bangkok. The selection of drug measurements for parent compounds (and carboxyprimaquine, the major metabolite of primaquine) are contingent on the availability of funding, with the analysis of methylene blue and primaquine/carboxyprimaquine levels being a priority above the analysis of other drugs in this study.

<u>*Cytochrome P450 (CYP) 2D6 and G6PD genotyping*</u>

Venous blood samples will be collected in EDTA and cryopreserved at – 80C for later analysis of P450 cytochrome polymorphisms by Thermo Fisher Scientific OpenArray Technology and Copy Number Variation (CNV) assays on the QuantStudio™ 12K Flex Real-Time PCR System, as well as genotyping for the known common polymorphisms for G6PD deficiency in western Africa. There samples will be tested at Radboud University or the University of Helsinki.

Data management and analysis

All data will be stored on secure password protected databases. Data that are collected on paper forms will be double entered. Data collected through hand held devices or directly produced by laboratory equipment will be examined for quality assurance and fed into the database using double data entry. <u>A full analysis plan will be developed before the trial starts and be reviewed by the DSMC.</u>

Ethical issues

This research will be conducted in compliance with the protocol, Good Clinical Practice (GCP) guidelines, and all applicable regulatory requirements. A copy of the protocol, informed consent forms, and any other documents given to study participants will be submitted to the ethics committees (EC) of all the institutions involved. Written approval will be obtained for all subsequent amendments to the protocol, informed consent documents, and other study documentation referenced above. The investigator will notify the ECs and DSMC of violations of the protocol and serious adverse events.

<u>*Subject information and consent*</u>

Consenting procedures will vary based on age of the potential participant. Participants aged 18 years and above will provide informed consent. For participants under 18 years of age we will seek parental consent. In addition to parental consent, assent will be sought for children aged 12-17 years.

The informed consent document will be used to explain the risks and benefits of study participation to the participant and in the case of participants < 18 years, the participant's parent in simple terms before the subject is enrolled in the study. The informed consent document contains a statement that the consent is freely given, that the subject is aware of the risks and benefits of entering the study, and that the subject is free to withdraw from the study at any time. Written consent must be given by the participant or the participant's parent, after the receipt of detailed information on the study. Written informed consent must be obtained from each participant or participant's parent prior to enrolment in the study. The informed consent form will be signed and personally dated by the participant or the participant's parent and the person who conducts the informed consent discussion. The original signed informed consent form will be retained in the participant's chart and another copy will be provided to him. A participant who is unable to read or write will place an imprint of his finger in place of a

signature; in addition, an independent witness will sign the consent form to attest that the information in the consent form was orally conveyed to the participant.

Confidentiality

The investigator will ensure that the subject's anonymity is maintained. Participants will not be identified in any publicly released reports of this study. All records will be kept confidential to the extent provided by laws and regulations. The study monitors and other authorized representatives of the regulatory authorities may inspect all documents and records required to be maintained by the Investigator.

All laboratory specimens, evaluation forms, reports, and other records that leave the site will be identified only by a coded number in order to maintain subject confidentiality.

Risks

There are minimal risks of hemolysis associated with the administration of single dose of 0.25 mg/kg of primaquine [49] or 15 mg/kg/day x 3 days of methylene blue in G6PDn males [36]. Several previous clinical trials with higher doses of primaquine and methylene blue have been conducted in younger age groups without any clinically significant side effects. Each participant will nevertheless be warned of the risks associated with primaquine and methylene blue use and will be monitored for 42 days. Participants will be asked to return to the clinic for reassessment should they feel unwell, feel out of breath, look pale, or notice their urine is a dark color. For methylene blue, urine will become bright blue or green for several days that can possibly dye clothing, and the sclera a faint blue color. This side effect of the treatment, which has no clinical consequences, will be explained in detail. Other side effects of primaquine and methylene blue include nausea, vomiting, loss of appetite, and stomach cramps, occasionally headaches and visual disturbance and pruritus.

While a recent study showed that SP-AQ is efficacious in clearing asymptomatic infections in Mali (study conducted in pregnant women [62]), given widespread drug resistance against SP and AQ in several other regions in Africa, there is a slight risk of treatment failure and parasitological relapse of falciparum malaria for participants receiving SP-AQ or SP-AQ-SLD PQ. Participants will be informed of this risk, and that if parasitological relapse occurs, they will be promptly treated with artemether-lumefantrine per Mali ministry of health guidelines.

In adults, the most common temporary side effects with DP are anemia, headache, QTc prolongation, tachycardia, asthenia and fever. Finger pricks and venipuncture are associated with pain and bruising at the site of the prick, and rarely infection.

Risks will be minimized by the study sites by ensuring adequate training of staff in all procedures and supply of consumables. The study site adheres to the standards of GCP and will be monitored.

Benefits

The benefits of the participation include free treatment of malaria and other diseases that may occur during the follow-up period. Participants who are in the groups that get the study medicine, primaquine or methylene blue, may be less infectious to mosquitoes and reduce the chance of having mosquitoes in their environment becoming infectious. Findings will help in the treatment of future patients and

communities with malaria and further optimize the design of community intervention programmes such as SMC.

Compensation

Participants will receive compensation for the time and travel for protocol specified visits. This compensation will be carefully evaluated and provided upon agreement of the local ethics committee. In Mali, the estimated cost of time and travel expense per visit in Ouelessebougou for residents of Ouelessebougou was estimated to 1000 F cfa (~2.4 US $). The compensation for enrolment visit and other visits during the first 8 hours including the hospitalization was estimated to 10000 F CFA (~24.0 US $) for participants ≥ 18 years of age and 5000 (~12.0 US $) for participants < 18 years of age that stay up to 12 hours.

Use and storage of study samples

Samples collected will be stored at MRTC in Bamako, Mali or the University of California San Francisco, USA, or both, and may also be shipped to collaborating research centers such as Radboud University medical center, the Mahidol Oxford Research Unit, and/or the London School of Hygiene & Tropical Medicine to perform specific tests as described above. Samples may be kept for a maximum of 10 years.

References

1. Gosling RD, Okell L, Mosha J, Chandramohan D: **The role of antimalarial treatment in the elimination of malaria.** *Clin Microbiol Infect* 2011, **17:**1617-1623.
2. Wilson AL, Taskforce IP: **A systematic review and meta-analysis of the efficacy and safety of intermittent preventive treatment of malaria in children (IPTc).** *PLoS One* 2011, **6:**e16976.
3. Maiga H, Djimde AA, Beavogui AH, Toure O, Tekete M, Sangare CP, Dara A, Traore ZI, Traore OB, Dama S, et al: **Efficacy of sulphadoxine-pyrimethamine + artesunate, sulphadoxine-pyrimethamine + amodiaquine, and sulphadoxine-pyrimethamine alone in uncomplicated falciparum malaria in Mali.** *Malar J* 2015, **14**:64.
4. Bukirwa H, Critchley J: **Sulfadoxine-pyrimethamine plus artesunate versus sulfadoxine-pyrimethamine plus amodiaquine for treating uncomplicated malaria.** *Cochrane Database Syst Rev* 2006**:**CD004966.
5. Cairns M, Roca-Feltrer A, Garske T, Wilson AL, Diallo D, Milligan PJ, Ghani AC, Greenwood BM: **Estimating the potential public health impact of seasonal malaria chemoprevention in African children.** *Nat Commun* 2012, **3:**881.
6. **WHO Policy Recommendation: Seasonal Malaria Chemoprevention (SMC) for Plasmodium falciparum malaria control in highly seasonal transmission areas of the Sahel sub-region in Africa** [http://www.who.int/malaria/publications/atoz/smc_policy_recommendation_en_032012.pdf?ua=1]
7. Bakyaita N, Dorsey G, Yeka A, Banek K, Staedke SG, Kamya MR, Talisuna A, Kironde F, Nsobya S, Kilian A, et al: **Sulfadoxine-pyrimethamine plus chloroquine or amodiaquine for uncomplicated falciparum malaria: a randomized, multisite trial to guide national policy in Uganda.** *Am J Trop Med Hyg* 2005, **72:**573-580.
8. Naidoo I, Roper C: **Mapping 'partially resistant', 'fully resistant', and 'super resistant' malaria.** *Trends in Parasitology* 2013, **29:**505-515.
9. Bojang K, Akor F, Bittaye O, Conway D, Bottomley C, Milligan P, Greenwood B: **A randomised trial to compare the safety, tolerability and efficacy of three drug combinations for intermittent preventive treatment in children.** *PLoS One* 2010, **5:**e11225.
10. Cisse B, Cairns M, Faye E, O ND, Faye B, Cames C, Cheng Y, M ND, Lo AC, Simondon K, et al: **Randomized trial of piperaquine with sulfadoxine-pyrimethamine or dihydroartemisinin for malaria intermittent preventive treatment in children.** *PLoS One* 2009, **4:**e7164.
11. Nankabirwa JI, Wandera B, Amuge P, Kiwanuka N, Dorsey G, Rosenthal PJ, Brooker SJ, Staedke SG, Kamya MR: **Impact of intermittent preventive treatment with dihydroartemisinin-piperaquine on malaria in Ugandan schoolchildren: a randomized, placebo-controlled trial.** *Clin Infect Dis* 2014, **58:**1404-1412.
12. Halliday KE, Okello G, Turner EL, Njagi K, Mcharo C, Kengo J, Allen E, Dubeck MM, Jukes MCH, Brooker SJ: **Impact of Intermittent Screening and Treatment for Malaria among School Children in Kenya: A Cluster Randomised Trial.** *Plos Medicine* 2014, **11**.
13. Bousema T, Okell L, Shekalaghe S, Griffin JT, Omar S, Sawa P, Sutherland C, Sauerwein R, Ghani AC, Drakeley C: **Revisiting the circulation time of Plasmodium falciparum gametocytes:**

molecular detection methods to estimate the duration of gametocyte carriage and the effect of gametocytocidal drugs. *Malar J* 2010, **9:**136.
14. Bousema JT, Schneider P, Gouagna LC, Drakeley CJ, Tostmann A, Houben R, Githure JI, Ord R, Sutherland CJ, Omar SA, Sauerwein RW: **Moderate effect of artemisinin-based combination therapy on transmission of Plasmodium falciparum.** *J Infect Dis* 2006, **193:**1151-1159.
15. Beshir KB, Sutherland CJ, Sawa P, Drakeley CJ, Okell L, Mweresa CK, Omar SA, Shekalaghe SA, Kaur H, Ndaro A, et al: **Residual Plasmodium falciparum parasitemia in Kenyan children after artemisinin-combination therapy is associated with increased transmission to mosquitoes and parasite recurrence.** *J Infect Dis* 2013, **208:**2017-2024.
16. Dicko A, Brown J, Diawara H, Baber I, Mahamar A, Soumare HM, Sanogo K, Koita F, Keita S, Traore SF, et al: **A randomized, controlled, dose-adaptive trial of primaquine to reduce transmission of P.**

falciparum malaria in Mali. *Submitted* 2015.
17. White NJ, Qiao LG, Qi G, Luzzatto L: **Rationale for recommending a lower dose of primaquine as a Plasmodium falciparum gametocytocide in populations where G6PD deficiency is common.** *Malar J* 2012, **11:**418.
18. Coulibaly B, Zoungrana A, Mockenhaupt FP, Schirmer RH, Klose C, Mansmann U, Meissner PE, Muller O: **Strong gametocytocidal effect of methylene blue-based combination therapy against falciparum malaria: a randomised controlled trial.** *PLoS One* 2009, **4:**e5318.
19. Adjalley SH, Johnston GL, Li T, Eastman RT, Ekland EH, Eappen AG, Richman A, Sim BK, Lee MC, Hoffman SL, Fidock DA: **Quantitative assessment of Plasmodium falciparum sexual development reveals potent transmission-blocking activity by methylene blue.** *Proc Natl Acad Sci U S A* 2011, **108:**E1214-1223.
20. Baird JK, Schwartz E, Hoffman SL: **Prevention and treatment of vivax malaria.** *Curr Infect Dis Rep* 2007, **9:**39-46.
21. White NJ: **Primaquine to prevent transmission of falciparum malaria.** *Lancet* 2013, **13:**175-181.
22. WHO: **World Malaria Report 2011.** *Geneva: World Health Organization, 2011*.
23. Arango EM, Upegui YA, Carmona-Fonseca J: **Efficacy of different primaquine-based antimalarial regimens against Plasmodium falciparum gametocytemia.** *Acta Trop* 2012, **122:**177-182.
24. Blair S, Lacharme LL, Fonseca JC, Tobon A: **[Resistance of Plasmodium falciparum to 3 antimalarials in Turbo (Antioquia, Colombia), 1998].** *Rev Panam Salud Publica* 2001, **9:**23-29.
25. Zongo I, Dorsey G, Rouamba N, Dokomajilar C, Sere Y, Rosenthal PJ, Ouedraogo JB: **Randomized comparison of amodiaquine plus sulfadoxine-pyrimethamine, artemether-lumefantrine, and dihydroartemisinin-piperaquine for the treatment of uncomplicated Plasmodium falciparum malaria in Burkina Faso.** *Clin Infect Dis* 2007, **45:**1453-1461.
26. Guttmann P, Ehrlich P: **Ueber die Wirkung des Methylenblau bei Malaria.** *Berliner Klin Wochenschr* 1891, **39:**953-956.
27. B. C, M. P, M. B, P.E. M, E. N, C. K, M. K, N. B-R, A. W, S.B. S, et al: **Efficacy and safety of triple combination therapy with artesunate-amodiaquine-methylene blue for falciparum malaria in children: a randomized controlled trial in Burkina Faso.** *J Infect Dis* 2015, **211:**689-697.

28. Delves MJ, Ruecker A, Straschil U, Lelievre J, Marques S, Lopez-Barragan MJ, Herreros E, Sinden RE: **Male and Female Plasmodium falciparum Mature Gametocytes Show Different Responses to Antimalarial Drugs.** *Antimicrobial Agents and Chemotherapy* 2013, **57:**3268-3274.
29. White NJ, Ashley EA, Recht J, Delves MJ, Ruecker A, Smithuis FM, Eziefula AC, Bousema T, Drakeley C, Chotivanich K, et al: **Assessment of therapeutic responses to gametocytocidal drugs in Plasmodium falciparum malaria.** *Malar J* 2014, **13:**483.
30. Chen IT, Gosling RD: **Targeting Plasmodium falciparum with primaquine: same efficacy, improved safety with a lower dose?** *Expert Rev Clin Pharm* 2014, **7:**681-686.
31. Meissner PE, Mandi G, Witte S, Coulibaly B, Mansmann U, Rengelshausen J, Schiek W, Jahn A, Sanon M, Tapsoba T, et al: **Safety of the methylene blue plus chloroquine combination in the treatment of uncomplicated falciparum malaria in young children of Burkina Faso [ISRCTN27290841].** *Malar J* 2005, **4:**45.
32. Meissner PE, Mandi G, Coulibaly B, Witte S, Tapsoba T, Mansmann U, Rengelshausen J, Schiek W, Jahn A, Walter-Sack I, et al: **Methylene blue for malaria in Africa: results from a dose-finding study in combination with chloroquine.** *Malar J* 2006, **5:**84.
33. Bountogo M, Zoungrana A, Coulibaly B, Klose C, Mansmann U, Mockenhaupt FP, Burhenne J, Mikus G, Walter-Sack I, Schirmer RH, et al: **Efficacy of methylene blue monotherapy in semi-immune adults with uncomplicated falciparum malaria: a controlled trial in Burkina Faso.** *Trop Med Int Health* 2010, **15:**713-717.
34. Mandi G, Witte S, Meissner P, Coulibaly B, Mansmann U, Rengelshausen J, Schiek W, Jahn A, Sanon M, Wust K, et al: **Safety of the combination of chloroquine and methylene blue in healthy adult men with G6PD deficiency from rural Burkina Faso.** *Trop Med Int Health* 2005, **10:**32-38.
35. Muller O, Mockenhaupt FP, Marks B, Meissner P, Coulibaly B, Kuhnert R, Buchner H, Schirmer RH, Walter-Sack I, Sie A, Mansmann U: **Haemolysis risk in methylene blue treatment of G6PD-sufficient and G6PD-deficient West-African children with uncomplicated falciparum malaria: a synopsis of four RCTs.** *Pharmacoepidemiol Drug Saf* 2013, **22:**376-385.
36. Coulibaly B PM, Bountogo M, Meissner PE, Nebié E, Klose C, Kieser M, Berens-Riha N, Wieser A, Sirima SB, Breitkreutz J, Schirmer RH, Sié A, Mockenhaupt FP, Drakeley C, Bousema T, Müller O: **Efficacy and Safety of triple combination therapy with artesunate-amodiaquine-methylene blue for falciparum malaria in children: a randomised controlled trial in Burkina Faso.** *J Infect Dis* 2015, **211:**689-697.
37. Dicko A, Diallo AI, Tembine I, Dicko Y, Dara N, Sidibe Y, Santara G, Diawara H, Conare T, Djimde A, et al: **Intermittent preventive treatment of malaria provides substantial protection against malaria in children already protected by an insecticide-treated bednet in Mali: a randomised, double-blind, placebo-controlled trial.** *PLoS Med* 2011, **8:**e1000407.
38. Cappellini MD, Fiorelli G: **Glucose-6-phosphate dehydrogenase deficiency.** *Lancet* 2008, **371:**64-74.
39. Howes RE, Battle KE, Satyagraha AW, Baird JK, Hay SI: **G6PD deficiency: global distribution, genetic variants and primaquine therapy.** *Adv Parasitol* 2013, **81:**133-201.
40. Eziefula AC, Pett H, Grignard L, Opus S, Kiggundu M, Kamya MR, Yeung S, Staedke SG, Bousema T, Drakeley C: **Glucose-6-phosphate dehydrogenase status and risk of hemolysis in Plasmodium falciparum-infected African children receiving single-dose primaquine.** *Antimicrob Agents Chemother* 2014, **58:**4971-4973.

41. Shekalaghe SA, ter Braak R, Daou M, Kavishe R, van den Bijllaardt W, van den Bosch S, Koenderink JB, Luty AJF, Whitty CJM, Drakeley C, et al: **In Tanzania, Hemolysis after a Single Dose of Primaquine Coadministered with an Artemisinin Is Not Restricted to Glucose-6-Phosphate Dehydrogenase-Deficient (G6PD A-) Individuals.** *Antimicrobial agents and chemotherapy* 2010**:**1762-1768.
42. Bousema T, Dinglasan RR, Morlais I, Gouagna LC, van Warmerdam T, Awono-Ambene PH, Bonnet S, Diallo M, Coulibaly M, Tchuinkam T, et al: **Mosquito feeding assays to determine the infectiousness of naturally infected Plasmodium falciparum gametocyte carriers.** *PLoS One* 2012, **7:**e42821.
43. **Single Low-dose Primaquine to Interrupt P. falciparum Transmission in Africa: A Roadmap Update. Meeting Summary.** [http://globalhealthsciences.ucsf.edu/sites/default/files/content/ghg/mei-london-pq-summary.pdf]
44. Mihaly GW, Ward SA, Edwards G, Nicholl DD, Orme ML, Breckenridge AM: **Pharmacokinetics of primaquine in man. I. Studies of the absolute bioavailability and effects of dose size.** *Br J Clin Pharmacol* 1985, **19:**745-750.
45. Ronn A, Bygbjerg I: **Unexpected high primaquine concentrations in acutely ill malaria patients.** *Lancet* 1993, **341:**305.
46. Cuong BT BV, Dai B, Duy DN, Lovell CM, Rieckmann KH, Edstein MD: **Does gender, food, or grapefruit juice alter the pharmacokinetics of primaquine in healthy subjects?** *Br J Clin Pharmacol* 2006, **61:**682-689.
47. Edwards G, Mcgrath CS, Ward SA, Supanaranond W, Yakamee SP, Davis TME, White NJ: **Interactions among Primaquine, Malaria Infection and Other Antimalarials in Thai Subjects.** *British Journal of Clinical Pharmacology* 1993, **35:**193-198.
48. **Eurartesim dosing guidelines** [http://www.ema.europa.eu/docs/en_GB/document_library/EPAR_-_Product_Information/human/001199/WC500118113.pdf]
49. Eziefula AC BT, Yeung S, Kamya M, Owaraganise A, Gabagaya G, Bradley J, Grignard L, Lanke KH, Wanzira H, Mpimbaza A, Nsobya S, White N J, Webb EL, Staedke SG, Drakeley C: **Single dose primaquine for clearance of Plasmodium falciparum gametocytes in children with uncomplicated malaria in Uganda: a randomised, controlled, double-blind, dose-ranging trial.** *Lancet Infect Dis* 2014, **14:**130-139.
50. WHO: **Guidelines for the treatment of malaria.** 2010.
51. **Co-artem dosing instructions** [https://www.pharma.us.novartis.com/product/pi/pdf/coartem.pdf]
52. Andrews L, Andersen RF, Webster D, Dunachie S, Walther RM, Bejon P, Hunt-Cooke A, Bergson G, Sanderson F, Hill AV, Gilbert SC: **Quantitative real-time polymerase chain reaction for malaria diagnosis and its use in malaria vaccine clinical trials.** *Am J Trop Med Hyg* 2005, **73:**191-198.
53. Schneider P, Schoone G, Schallig H, Verhage D, Telgt D, Eling W, Sauerwein R: **Quantification of Plasmodium falciparum gametocytes in differential stages of development by quantitative nucleic acid sequence-based amplification.** *Mol Biochem Parasitol* 2004, **137:**35-41.
54. Joice RC NV, Montgomery J, Seydel KB, Pierre-Louis W, et al. : **Simultaneous Quantification of Asexual and Sexual Stages During Malaria Infection** In *ASTMH Philadelphia*2011.

55. Felger I, Maire M, Bretscher MT, Falk N, Tiaden A, Sama W, Beck HP, Owusu-Agyei S, Smith TA: **The dynamics of natural Plasmodium falciparum infections.** *PLoS One* 2012, **7:**e45542.
56. Daniels R, Chang HH, Sene PD, Park DC, Neafsey DE, Schaffner SF, Hamilton EJ, Lukens AK, Van Tyne D, Mboup S, et al: **Genetic Surveillance Detects Both Clonal and Epidemic Transmission of Malaria following Enhanced Intervention in Senegal.** *Plos One* 2013, **8**.
57. Karl S, David M, Moore L, Grimberg BT, Michon P, Mueller I, Zborowski M, Zimmerman PA: **Enhanced detection of gametocytes by magnetic deposition microscopy predicts higher potential for Plasmodium falciparum transmission.** *Malar J* 2008, **7:**66.
58. Karl S, Davis TM, St-Pierre TG: **A comparison of the sensitivities of detection of Plasmodium falciparum gametocytes by magnetic fractionation, thick blood film microscopy, and RT-PCR.** *Malar J* 2009, **8:**98.
59. Taylor LH, Walliker D, Read AF: **Mixed-genotype infections of the rodent malaria Plasmodium chabaudi are more infectious to mosquitoes than single-genotype infections.** *Parasitology* 1997, **115:**121-132.
60. Tao DY, Ubaida-Mohien C, Mathias DK, King JG, Pastrana-Mena R, Tripathi A, Goldowitz I, Graham DR, Moss E, Marti M, Dinglasan RR: **Sex-partitioning of the Plasmodium falciparum Stage V Gametocyte Proteome Provides Insight into falciparum-specific Cell Biology.** *Molecular & Cellular Proteomics* 2014, **13:**2705-2724.
61. **G6PD deficiency Care Start 3 insert.** (Access Bio I ed.
62. Coulibaly SO, Kayentao K, Taylor S, Guirou EA, Khairallah C, Guindo N, Djimde M, Bationo R, Soulama A, Dabira E, et al: **Parasite clearance following treatment with sulphadoxine-pyrimethamine for intermittent preventive treatment in Burkina-Faso and Mali: 42-day in vivo follow-up study.** *Malar J* 2014, **13:**41.
